# Illustration of a process for the calculation and validation of minimum dietary diversity indicators using an existing dataset of 2 to younger than 10-year-old children

**DOI:** 10.1017/S0007114525000807

**Published:** 2025-05-14

**Authors:** Johanna H. Nel, Nelia P. Steyn, Marjanne Senekal

**Affiliations:** 1 Department of Logistics, University of Stellenbosch, Stellenbosch, South Africa; 2 Department of Human Biology, Health Sciences Faculty, University of Cape Town, Cape Town, South Africa

**Keywords:** Minimum dietary diversity indicator, Dietary diversity score, Micronutrient adequacy, Performance criteria, Sensitivity, Specificity

## Abstract

This study aims to illustrate a process approach for the calculation of minimum dietary diversity (MDD) indicators for interpretation of dietary diversity (DD) scores and to validate the MDD indicator as a proxy for adequate micronutrient intake using an existing dataset for 2 to younger than 10-year-old South African children. The DD scores were derived from nine food groups, adjusted from the ten food groups for women of reproductive age by combining pulses, nuts and seeds. Three reference methods were used to inspect micronutrient adequacy, namely the mean adequacy ratio and the mean probability of adequacy (MPA) using a single 24-h recall, and the MPA derived from usual intake using more than one 24-hour recall in a sub-sample. Adequacy threshold levels and candidate MDD indicators were inspected and validated using several performance criteria. Results show that the mean and median DD scores were 3·6 and 3·1, respectively. The resulting MDD indicators varied between 3 and 4 out of nine food groups favouring the identification of children with adequate and inadequate intake, respectively, depending on the method used and the age group. Our results and those from others furthermore support a simplified method or ‘rule of thumb’ for the determination of an MDD indicator to establish the integer values below and above the median of the DD scores. We conclude that finding a valid MDD indicator can be done using different methodologies and that results underscore the potential of a simplified method for determining an MDD indicator.

Adequate dietary intake assessment underpins nutritional profiling of individuals, communities and populations to advise on issues such as care, intervention, decision making and policy development. However, comprehensive assessment of usual dietary intake is cumbersome and costly^(1)^. This has resulted in the investigation of alternative simplified options for screening dietary adequacy of individuals and populations such as dietary diversity (DD) scores and minimum dietary diversity (MDD) indicators for interpretation of DD scores^(1–6)^.

The DD concept was first suggested by Guthrie and Sheer^(3)^ and is based on the premise that a diet lacking in diversity can increase the risk of micronutrient deficiencies^(7,8)^. The underpinning assumption of DD assessment is that the higher the DD score the more likely it is that micronutrient intake of an individual or population is sufficient. A major advantage of DD assessment is that it can be calculated from a single quantified 24-h recall, or frequency of intake of specific food groups without necessarily quantifying intake as such^(7,8)^.

Variations in DD assessment that are apparent from the literature include recommendations on the minimum amount of food from a food group that should be consumed to be considered in the calculation of a DD score, the number of food groups to be considered in this calculation and the type of foods to be included in each of the specified food groups^(6)^. Versions/definitions of DD food groups initially included either four groups (milk, meat, fruits and vegetables and breads and cereals)^(3,9)^ or five food groups (dairy, grain, fruits, vegetables and fleshy foods)^(4,10–13)^. Hatløy *et al.* ^(1)^ increased the number of food groups to eight (starchy staples, vegetables, milk, meat, fish, egg, fruits and green leaves). Several studies used nine food groups, adapted from food groups based on the outcome of discussions held at a workshop in Rome in October 2004^(14)^. These nine food groups are starchy staples, vitamin A-rich fruit and vegetables, other fruit, other vegetables, legumes and nuts, fats and oils, meat/poultry/fish, dairy and eggs^(5,15,16)^. As is evident from previous food groupings, some researchers included a fat and oil group. This has been challenged as fats and oils are mostly energy dense and micronutrient poor and could over-inflate the nutrient adequacy outcomes^(15,17–19)^.

The most recent two internationally recognised DD food group definitions are firstly that by the WHO/UNICEF for children 6 to younger than 24 months that specify eight groups (breast milk, grains, roots and tubers; legumes, nuts and seeds; dairy products; flesh foods; eggs; vitamin A-rich fruits and vegetables and other fruits and vegetables), with an associated MDD indicator for infant and young child feeding of five food groups^(7)^. The second food group definition is by the FAO for adult women of reproductive age, where the DD score is calculated from ten food groups (grains, roots and tubers; pulses; nuts and seeds; dairy; flesh foods; eggs; dark green leafy vegetables; other vitamin A-rich fruits and vegetables; other vegetables; other fruit), with an associated MDD indicator for women of reproductive age (MDD-W) of five food groups^(8)^.

The challenge encountered with the application of DD scores in the assessment of micronutrient intake is the establishment of thresholds reflecting inadequate *v*. adequate intake. The validity of DD assessment as a proxy for nutrient adequacy was first tested using regression techniques and correlation analyses by Krebs-Smith *et al.* ^(10)^, among others^(12,20–22)^. Schuette *et al.* ^(12)^ were the first to inspect the relationship between an MDD indicator and adequate dietary intake using sensitivity and specificity analysis for the interpretation of the DD scores. Hatløy *et al.* ^(1)^ tested DD scores below specific cut-off points to find the maximum DD score that would identify the proportion with a low mean nutrient adequacy (MAR) but with a high sensitivity without losing too much specificity. Hatløy *et al.* ^(1)^ proposed an MAR of 0·75 (75 %) as a threshold for a nutritional inadequate diet, similar to Schuette *et al.* ^(12)^. The motivation given by Schuette *et al.* ^(12)^ for this threshold was that an adequacy threshold of 75 % was less liberal than 67 % of the recommended dietary allowance, but not as stringent as 100 % of the recommended dietary allowance.

A further approach of validating MDD indicators was outlined by Arimond *et al.* ^(6,23)^ that involved the determination of a mean probability of adequacy (MPA) for a population using usual intakes, validated by food group diversity indicators. In these studies, 24-h recall data, adjusted for day-to-day variation for eleven micronutrients using data from additional recalls in a subsample, were used in combination with the estimated average requirements in the population. The sensitivity of different MDD indicators was tested against several adequacy threshold levels of the MPA.

It is important to consider that the above-mentioned MDD indicators recommended by the WHO/UNICEF^(7)^ for infants and young children and by the FAO^(8)^ for women may not be applicable in all settings and for children older than 6 years. For example, in South Africa bread and maize flour are fortified with eight micronutrients including Fe, Zn, vitamin A, thiamine, riboflavin, niacin, vitamin B_6_ and folate^(24)^. These eight micronutrients are provided by two food items, which would not naturally include the majority of these nutrients. Consumption of the fortified foods may thus reduce the number of food groups needed to consume adequate amounts of micronutrients, and thus potentially change the MDD indicator that would reflect good DD (and micronutrient intake) with acceptable sensitivity and specificity in this country. This may impact the application and interpretation of DD scores in different settings.

This study aims to illustrate a process approach for the calculation of MDD indicators to interpret DD scores and to validate the MDD indicators as a proxy for adequate intake using an existing dataset of 2 to younger than 10-year-old South African children.

## Methods

### Study design

**Figure 1.**
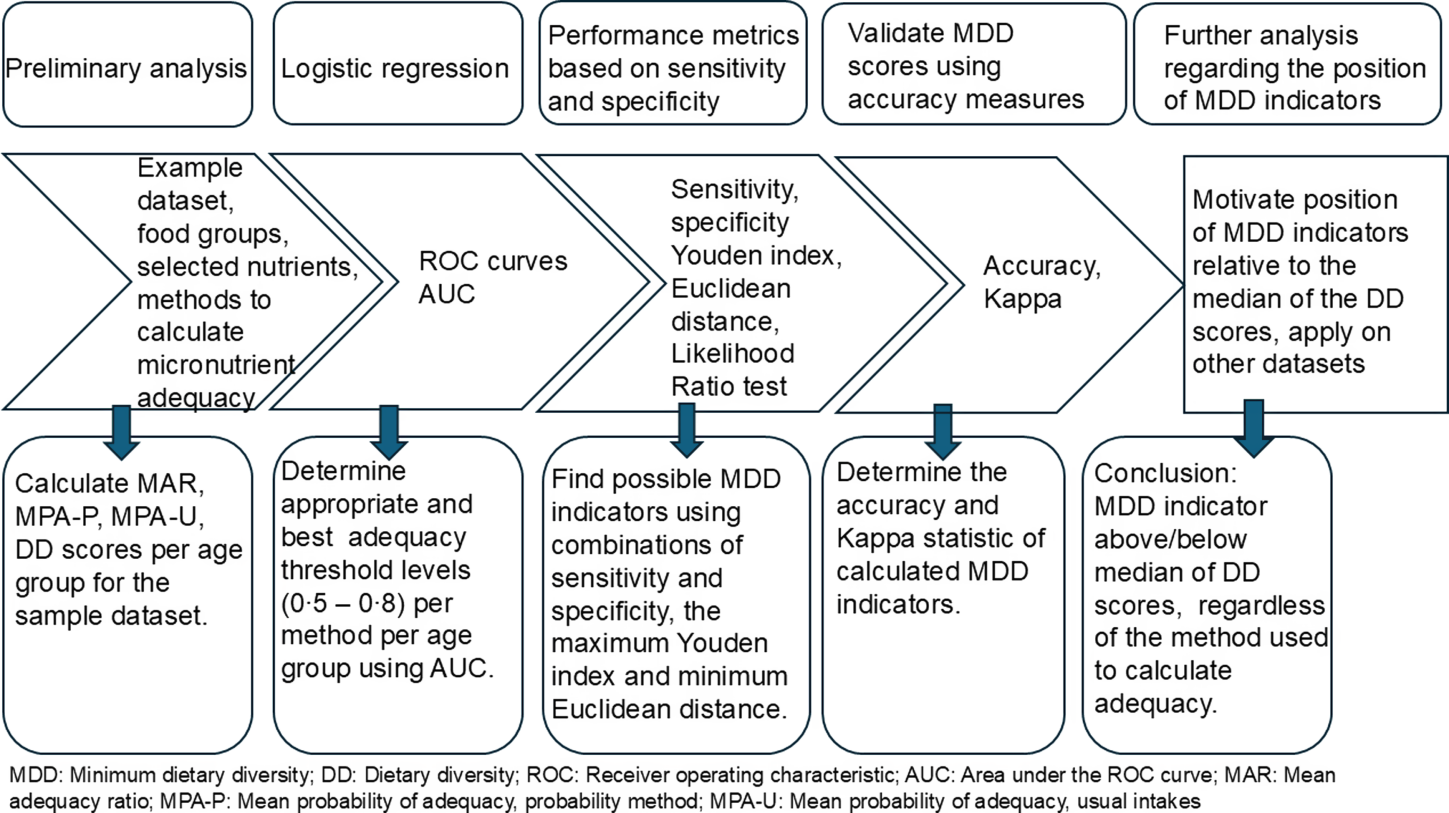
Diagram to illustrate the process followed for the calculation and validation of minimum dietary diversity indicators using an existing dataset of 2 to younger than 10-year-old children.

The process followed in this research is illustrated in Fig. 1. The first step involved calculation of nutrient adequacy measures namely MAR, mean probability of adequacy calculated using the probability method (MPA-P) and MPA-usual. (Background details on these methods are provided in the online supplementary material (Supplementary S.1).) Although it is generally acknowledged that using dietary intake methods where within-person variance is considered, many researchers are still forced to make use of a single 24-h recall^(17,18,25–28)^, and we therefore included the older methods using MAR and MPA-P in our analyses. The second step involved determination of the most appropriate adequacy threshold using logistic regression to obtain receiver operating characteristic (ROC) curves and the AUC. The third step involved assessing performance of various possible MDD indicators using the following criteria: sensitivity and specificity, the maximum Youden index, minimum Euclidean distance and likelihood ratio test. The fourth step focused on validation of the MDD indicators decided upon in the third step, by calculating accuracy levels and the Kappa statistic. The final step involved the motivation for the final recommended MDD indicator using results from this study integrated with results published by others, for example Arimond *et al.* ^(23)^, Rani^(29)^, Caswell *et al.* ^(30)^, Diop *et al.* ^(31)^ and Monge-Rojas *et al.* ^(32)^.

### Dataset

This study used data from the 2018 Provincial Dietary Data Intake Study of children 2 to <10- year-old (*n* 1170) from two provinces in South Africa, Gauteng and the Western Cape. These are the most rapidly urbanising and wealthiest provinces, with extensive migration from rural areas to cities in search of employment and better quality of life^(33)^. In this study, analyses were conducted in two age groups, namely 2 to <6 years (*n* 691) and 6 to <10 years (*n* 479). A single 24-h recall was obtained from the total sample, while two additional 24-h recalls were obtained from representative sub-samples of 148 and 146, respectively. A detailed description of the PDIS study can be found in Senekal *et al.* ^(33)^.

### Food groups and nutrients selected for dietary diversity assessment

#### Food groups

As there are currently no international recommendations regarding food grouping for DD assessment for children 2 to <18-year-old food intake of the dataset was grouped using an adapted version of the ten defined food groups suggested by the WHO^(8)^ (MDD-W food groups) as outlined in the introduction. After examining a South African dietary intake study on commonly consumed foods^(34)^, as well as dietary intake results from the PDIS study,^(33)^ it was decided to combine pulses and nuts and seeds (Groups 2 and 3 of the food groups associated with the MDD-W) since foods in the nuts and seeds group were consumed by less than 10 % of children, resulting in an adjusted food grouping with nine food groups, referred to in this study as the SA-Child food groups.

The FAO^(8)^ guide for assessment of DD indicates that an intake of a minimum of 15 g from a food group could be set when considering inclusion of the food group in a DD score, but also mentions that this is not compulsory. The UNICEF infant and young child feeding guide^(7)^ concurs that setting a minimum intake in young children is not a requirement, indicating that non-quantified 24-h frequency of intake data is acceptable and adequate for DD assessment. For the purposes of this paper, we did not set a minimum intake level for a food to contribute to the DD score.

#### Nutrients

Fifteen nutrients were considered in the calculation of nutrient adequacy values, namely Ca, phosphorus, Fe, Zn, vitamin A, folate, thiamine, riboflavin, niacin, vitamin B_6_, vitamin B_12_, vitamin C, Mg, pantothenic acid and vitamin E. The fifteen nutrients were selected based on combinations of earlier studies^(5,6,19,23,35)^.

### Methods used for calculation of micronutrient intake adequacy

***Method 1*** is an adaptation from three fixed cut-off point methods described by Hatløy *et al.* ^(1)^; Steyn *et al.* ^(5)^ and Oldewage-Theron and Kruger^(19)^ to calculate nutrition adequacy ratios for each of the fifteen nutrients and the MAR using the single 24-h recall from the dataset for the children 2 to <10 years (*n* 1170). For this calculation, the nutrient intakes for each nutrient were divided by the estimated average requirements or adequate intake if the EAR was not available^(36)^ and were truncated at one if the ratio was greater than one^(37)^. Of note is that the EAR for Zn recommended by the FAO/WHO^(38)^ was used as suggested by Gibson & Ferguson^(39)^, and Allen *et al.* ^(40)^ for diets containing zinc with a high bioavailability, for example a diet high in foods fortified with Zn (online Supplementary S.2). Bread and maize meal that are fortified with Zn in South Africa are some of the most commonly consumed foods among children^(33,34)^.

***Method 2*** is an application of the probability approach described by Foote *et al.* ^(4)^ that involves the calculation of the probability of adequate intake of a nutrient. For these purposes the single 24-h recall from the existing dataset for the total representative sample was used (*n* 1170). According to this method, the probability that a given nutrient intake is adequate for an individual can be calculated if the requirement distribution is known. If this distribution is approximately normal, it is defined by the EAR as the mean value as well as the sd, calculated as the product of the CV and the EAR, divided by 100^(41)^. The CV values used were 15 % for niacin and 25 % for Zn, 20 % for vitamin A and 10 % for the rest^(16,41)^. Because the sample was representative, the underlying assumption was that all the nutrients, except for Ca and Fe, would have a normal distribution. Using the assumed normal distributions, the ‘PROBNORM’ function in SAS (SAS Institute Inc., Cary, NC, USA) was used to calculate the probability of adequacy of a specific nutrient intake using the probability method (PA-P), reflecting the proportion of the population with an intake that is less than the EAR (online Supplementary S.3). The resulting values for the PA-P range, by definition, from 0·0 to 1·0. The EAR for Zn was used assuming high bioavailability, as described by Gibson & Ferguson^(39)^ (online Supplementary S.1). The calculation of the PA-P for Ca (online Supplementary S.4) and Fe (online Supplementary S.5) was done using techniques described by Foote *et al.* ^(4)^. Distribution for Fe incorporated high bioavailability, as described by Gibson and Ferguson^(39)^. The MPA-P for this method, referred to as Method 2, is calculated as the mean value of the PA-Ps of the fifteen nutrients.

***Method 3*** involved calculation of the probability of adequate intake using usual intakes (PA-U) and the mean probability of adequate intake using usual intakes (MPA-U) for the fifteen nutrients by applying the EAR cut-point method to usual intakes as described by Arimond *et al.* ^(6,23)^. Usual intake was derived from the single 24-h recall from the dataset for the total representative sample intake plus additional intakes for two sub-samples. Steps taken to calculate the PA-Us and the MPA-U using Method 3 are provided in online Supplementary S.6. Etimated average requirements used are from the Institute of Medicine^(37)^ (online Supplementary S.3), Ca distributions from Foote *et al.* ^(4)^ (online Supplementary S.4), EAR Zn high bioavailability^(39)^ (online Supplementary S.2) and the probability distribution for Fe (high bioavailability) was as described by Gibson and Ferguson^(39)^ (online Supplementary S.5).

### Threshold levels

Threshold levels for adequate intake that ranged from 0·5 to 0·8, recommended by Martin–Prevel^(42)^, were investigated for each reference method calculating MAR, MPA-P and MPA-U, respectively. Also, if the number of children with mean adequate intake less than the adequacy threshold level is less than or equal to 10, the threshold level in question is not considered^(42)^. Furthermore, these threshold levels have been shown to be reasonable choices to define a positive indicator^(42)^.

For each reference method, we ran a logistic regression with the adequacy threshold levels as dependent variable and the DD scores as independent variable, to derive odds ratios and the AUC, which summarises the predictive power of the DD scores over all possible cut-offs, or potential MDD indicators. In the present study, the ‘best’ threshold levels for MAR, MPA-P and MPA-U were selected using the best AUC, as suggested by Prevel *et al.* ^(42)^. In general, an AUC of 0·5 suggests DD scores with no discrimination value, 0·5 to 0·7 with a fail to poor value, while 0·7 to 0·8 is considered fair, 0·8 to 0·9 is considered to be good and more than 0·9 is considered to be excellent^(43)^. An AUC cut-off of 0·7 was considered by Arimond *et al.* ^(44)^ as being acceptable for evaluating the proposed MDD indicators. A *χ* ^2^ test, testing that AUC = 0·5, was included to test the significance of the AUC.

### Performance metrics used to determine possible MDD indicators

For each reference method, a range of performance metrics, including sensitivity, specificity, the maximum Youden index, the minimum Euclidean distance and the likelihood ratio test were used to determine the MDD indicator for different adequacy threshold levels^(45)^.

The Youden index (J), the Euclidean distance (D) and the likelihood ratio test (LR) are calculated as follows^(43,45)^:

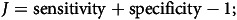




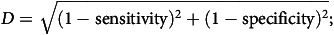




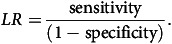




These measures are functions of sensitivity and specificity and are used to evaluate the performance of each possible MDD indicator per adequacy threshold level. For each possible MDD indicator, the sensitivity and specificity should be at least 0·6 and will still be considered if only one of the two is at least 0·5^(42)^. Also, higher values of the Youden index, and lower values for the Euclidean distance, both varying between 0 and 1, reflect good performance of a possible MDD indicator, for a given adequacy threshold level. The likelihood ratio (LR) test can be used to evaluate by how much a given MDD indicator will raise or lower the pretest probability of the threshold levels for adequacy^(45,46)^. Likelihood ratios essentially combine the benefits of both sensitivity and specificity into one index^(45)^.

### Validation of results using accuracy measures

Determining the accuracy measure and the measure of agreement between potential MDD indicators and adequacy threshold levels employ methodologies that do not include sensitivity and specificity directly and can be used to validate associations between resulting MDD indicators (using sensitivity and specificity related techniques) and adequacy threshold levels. Accuracy and agreement measures will also contribute to the identification of a final MDD-indicator^(42)^. The accuracy rate should be at least 0·7, and an accuracy rate of 0·6 will still be considered to establish an appropriate MDD indicator^(23,42)^. The accuracy rate is calculated as follows:

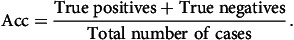




Additionally, the level of agreement between the resulting MDD indicator and the adequacy measures was assessed using the Cohen’s Kappa statistic^(25,26)^. The Kappa scores are interpreted as follows: poor agreement (<0·00), slight agreement (0·00–0·20), fair agreement (0·21–0·40), moderate agreement (0·41–0·60), substantial agreement (0·61–0·80) and almost perfect agreement (0·81–1·00)^(25,26)^.

### Ethics

The PDIS study was approved by the University of Cape Town Faculty of Health Sciences Human Research Ethics Committee (HREC REF: 326/2018). Parents or primary caregivers of children provided informed, signed consent. Additionally, children aged 6 to <10 years provided verbal assent. The study was conducted in accordance with the principles of the 2013 Declaration of Helsinki, Good Clinical Practice and the laws of South Africa^(47)^.

## Results

Table 1 presents the percentage of the PDIS sample that consumed at least one item from each of the nine SA-Child food groups adapted from the ten FAO food groups^(8)^. The mean (95 % CI of the mean) and median (95 % CI of the median) values of the DD scores were 3·6 (3·5, 3·7) and 3·1 (3·0, 3·2), respectively, for children aged 2 to <10 years, but the mean and median values per age group are also 3·6 and 3·1, respectively. Results in Table 1 show that starchy staples (almost 100 %), flesh foods (more than 80 %) and dairy (almost two-thirds) are the most consumed food groups amongst both age groups. Dark green vegetables, eggs and the combination of legumes, nuts and seeds are amongst the least consumed food groups.

**Table 1.** Percentage (95 % CI for the percentage) of the Provincial Dietary Intake Survey sample consuming the nine SA-Child food groups, by age

Food group	2–<6 years* (*n* 691)		6–<10 years* (*n* 479)		2–<10 years* (*n* 1170)		All† % of total energy intake (*n* 1170)	All† Per capita quantity consumed (*n* 1170)	
	Percentage	95 % CI	Percentage	95 % CI	Percentage	95 % CI		Mean (g)	95 % CI
1. Starchy staples	99·3	98·6, 100·0	99·7	99·1, 100·0	99·5	99·0, 100·0	40·6 %	368·9	349·1, 388·8
2. Legumes, nuts and seeds	12·3	8·8, 15·9	13·8	10·2, 17·4	13·0	10·6, 15·4	1·3 %	14·1	10·9, 17·3
3. Dairy	64·6	58·5, 70·6	59·4	52·7, 66·0	62·3	57·4, 67·2	5·8 %	114·5	100·8, 128·3
4. Flesh foods	81·0	76·9, 85·2	88·2	84·3, 92·2	84·2	81·5, 86·9	12·9 %	82·4	77·5, 87·3
5. Eggs	12·2	8·5, 16·0	11·4	7·7, 15·0	11·9	9·0, 14·8	1·2 %	9·7	7·4, 12·1
6. Dark green vegetables	7·6	5·0, 10·1	6·4	3·4, 9·4	7·0	5·0, 9·1	0·3 %	6·4	4·2, 8·6
7. Vitamin A rich fruit and vegetables‡	16·7	12·3, 21·1	13·0	9·2, 16·8	15·1	11·7, 18·5	0·5 %	11·2	8·3, 14·1
8. Other vegetables	33·2	29·0, 37·3	39·7	34·7, 44·7	36·0	32·2, 39·8	1·4 %	28·8	24·3, 33·4
9. Other fruit‡	35·5	29·5, 41·5	32·2	25·6, 38·9	34·1	29·4, 38·8	2·1 %	50·5	41·8, 59·1
DD score									
Mean (95 % CI)	3·6	3·5, 3·8	3·6	3·5, 3·8	3·6	3·5, 3·7			
Median (95 % CI)	3·1	2·9, 3·2	3·1	2·9, 3·3	3·1	3·0, 3·2			

SA Child Food groups = Group 1. Grains, roots and tubers, Group 2. Pulses, nuts and seeds, Group 3. Milk and milk products, Group 4. Meat, poultry and fish, Group 5. Eggs, Group 6. Dark leafy green vegetables, Group 7. Other vitamin A-rich fruits and vegetables, Group 8. Other vegetables and Group 9. Other fruits.

DD, Dietary diversity; CI, Confidence interval.

* Analysis done using complex survey design, weighted analyses.

† Percentage of total kilojoule intake for other food items not in the above groups is 33·9 %. The mean (95 % CI) per capita intake (g) of other food items is 337·2 (314·4, 360·0).

‡ As per WHO/UNICEF definition, fruit juices were included in the other (sugar sweetened beverages) and not in food groups 7 or 9^(7)^.

Table 2 shows the mean (95 % CI of the mean) and median (95 % CI of the median) intake for each of the nutrients included in the calculation of the adequacy measures, MAR, MPA-P and MPA-U. Descriptive statistics for the truncated NAR calculated using Method 1 and PA-P and PA-U calculated using Methods 2 and 3, respectively, per nutrient, by age group are also presented. Of note is that low adequacies (<0·50) were evident for calcium and pantothenic acid, especially when using Methods 2 and 3. If a threshold for adequacy of <0·70 is considered arbitrarily, vitamins C, E and B_12_ would also be categorised as inadequate.

**Table 2.** Mean* (95 % CI of mean) and median (95 % CI of median), as well as truncated nutrient adequacy ratio (NAR) calculated using Method 1 and probability of adequacy (PA-P and PA-U) calculated using Methods 2 and 3, respectively, per nutrient, by age group

Nutrient EAR or alternative reference value as specified	Age 2–<6 years (*n* 691)						Age 6–<10 years (*n* 479)					
	Mean (95 % CI of mean) Median (95 % CI of median)						Mean (95 % CI of mean) Median (95 % CI of median)					
	Truncated NAR (Method 1)		PA-P (Method 2)		PA-U (Method 3)		Truncated NAR (Method 1)		PA-P (Method 2)		PA-U (Method 3)	
	Percentage	95 % CI	Percentage	95 % CI	Percentage	95 % CI	Percentage	95 % CI	Percentage	95 % CI	Percentage	95 % CI
Calcium† (mg) EAR (Method 1): 2–3-yrs = 500 mg; 4–8 yrs = 800 mg; 9–<10 yrs = 1300 mg	359·0 (325·3, 392·7) 292·1 (253·6, 330·6)						352·2 (325·2, 379·2) 299·9 (270·1, 329·7)					
	51·3	47·6, 55·1	40·9	36·8, 45·0	39·5	35·9, 43·1	40·1	37·1, 43·2	27·9	24·6, 31·1	25·9	23·1, 28·2
Iron‡ (mg) EAR (Method 1): 2–3 yrs = 3·0 mg; 4–8 yrs = 4·1 mg; M:9–<10 yrs = 5·9 mg F:9–<10 yrs = 5·7 mg	8·5 (8·0, 8·9) 8·1 (7·6, 8·7)						10·6 (10·1, 11·1) 9·7 (9·3, 10·2)					
	99·5	99·2, 99·8	84·4	82·3, 86·5	88·3	86·7, 89·8	99·4	98·9, 99·8	87·4	85·7, 89·0	89·0	87·8, 90·1
Magnesium (mg) 1–3-yrs = 65 mg; 4–8 yrs = 110 mg; 9–<10 yrs = 200 mg	169·1 (159·9, 178·3) 159·9 (149·5, 170·2)						197·5 (189·9, 205·1) 188·8 (179·7, 197·9)					
	97·6	96·7, 98·5	88·5	85·5, 91·5	93·5	91·4, 95·5	95·6	94·2, 97·0	81·3	77·5, 85·1	84·7	81·4, 87·9
Phosphorus (mg) 1–3-yrs = 380 mg; 4–8 yrs = 405 mg; 9–<10 yrs = 1055 mg	599·9 (565·2, 634·5) 533·0 (487·1, 578·9)						698·1 (662·8, 733·4) 667·0 (622·8, 711·1)					
	93·6	92·1, 95·1	73·5	68·7, 78·4	82·2	78·5, 85·9	91·2	89·4, 93·0	72·5	68·2, 76·8	75·8	71·9, 79·8
Zinc‡ (mg) 1–3 yrs = 2·2 mg 4–8 yrs = 2·4 mg 9–10 yrs = 2·4 mg	7·0 (6·7, 7·4) 6·4 (5·9, 7·0)						8·5 (8·1, 8·9) 7·9 (7·4, 8·4)					
	99·8	99·6, 100·0	98·1	97·3, 99·0	99·8	99·5, 100·0	99·9	99·8, 100·0	98·7	98·0, 99·4	99·9	99·7, 100·0
Vitamin A (ug) 1–3 yrs = 210 ug 4–8 yrs = 275 ug M: 9–<10 yrs = 445 ug F: 9–<10 yrs = 420 ug	594·7 (514·4, 675·0) 379·0 (346·0, 412·1)						694·3 (577·2, 811·4) 431·2 (384·1, 478·6)					
	92·1	90·0, 94·2	76·1	71·9, 80·2	83·4	80·2, 86·6	90·1	87·8, 92·5	70·1	65·9, 74·3	75·6	71·6, 79·7
Vitamin C (mg) 1–3 yrs = 13 mg; 4–8 yrs = 22 mg; 9–<10 yrs = 39 mg	41·7 (35·8, 47·6) 24·1 (20·9, 27·3)						43·6 (36·1, 51·1) 27·3 (23·9, 30·7)					
	80·9	77·1, 84·6	65·1	59·8, 70·3	68·3	63·0, 73·5	78·9	74·8, 83·0	58·5	52·4, 64·7	58·5	52·4, 64·5
Vitamin E (mg) 1–3 yrs = 5 mg 4–8 yrs = 6 mg; 9–<10 yrs = 9 mg	7·9 (7·3, 8·6) 5·8 (5·5, 6·2)						11·0 (9·9, 12·1) 8·2 (7·4, 9·1)					
	81·1	78·4, 83·8	54·1	50·3, 58·0	58·2	54·5, 61·9	86·0	83·6, 88·4	64·5	59·4, 69·5	64·8	60·0, 69·7
Folate (ug) 1–3 yrs = 120 ug; 4–8 yrs = 160 ug; 9 yrs = 250 ug	243·6 (222·9, 264·3) 199·3 (180·0, 218·6)						284·6 (268·8, 300·3) 242·9 (231·9, 254·0)					
	92·4	90·6, 94·3	73·8	68·9, 78·6	80·2	75·8, 84·6	92·0	90·5, 93·6	72·7	68·9, 76·6	76·9	73·1, 80·7
Niacin (mgNE) 1–3 yrs = 5·0 mgNE; 4–8 yrs = 6·0 mgNE; 9–<10 yrs = 9·0 mgNE	13·6 (13·0, 14·2) 12·7 (11·8, 13·6)						17·3 (16·4, 18·2) 16·7 (15·6, 17·7)					
	98·4	97·8, 99·0	92·5	90·6, 94·4	97·5	96·4, 98·5	98·6	97·7, 99·4	94·0	91·8, 96·2	96·9	95·3, 98·5
Pantothenic acid 1–3-yrs = 2 mg; 4–8 yrs = 3 mg; 9–<10 yrs = 4 mg	3·4 (3·2, 3·6) 3·1 (2·8, 3·4)						3·5 (3·3, 3·8) 2·8 (2·5, 3·2)					
	85·5	83·1, 87·8	62·0	57·3, 66·8	65·1	60·4, 69·9	77·4	73·9, 80·9	47·3	41·0, 53·6	47·4	41·4, 53·3
Riboflavin (mg) 1–3 yrs = 0·4 mg; 4–8 yrs = 0·5 mg; 9–<10 yrs = 0·8 mg	0·9 (0·8, 0·9) 0·8 (0·7, 0·8)						1·0 (0·9, 1·1) 0·9 (0·8, 0·9)					
	93·9	92·1, 95·6	79·4	75·2, 83·7	86·8	83·4, 90·3	93·5	91·6, 95·3	76·3	71·5, 81·1	81·6	77·8, 85·4
Thiamine (mg) 1–3 yrs = 0·4 mg; 4–8 yrs = 0·5 mg; 9–<10 yrs = 0·7 mg	1·0 (1·0, 1·1) 0·9 (0·9, 1·0)						1·2 (1·1, 1·2) 1·1 (1·0, 1·2)					
	98·6	98·0, 99·2	93·2	90·9, 95·5	96·9	95·5, 98·3	98·5	97·5, 99·4	94·1	91·5, 96·7	96·4	94·5, 98·3
Vitamin B_6_ (mg) 1–3 yrs = 0·4 mg; 4–8 yrs = 0·5 mg; 9–<10 yrs = 0·8 mg	1·7 (1·6, 1·8) 1·6 (1·5, 1·7)						2·5 (2·3, 2·6) 2·2 (2·1, 2·4)					
	99·5	99·1, 99·8	97·7	96·3, 99·1	99·7	99·4, 100·0	99·8	99·5, 100·0	98·9	98·0, 99·8	99·6	99·0, 100·0
Vitamin B_12_ (ug) 1–3 yrs = 0·7 ug; 4–8 yrs = 1·0 ug; 9–<10 yrs = 1·5 ug	3·0 (2·4, 3·6) 1·3 (1·1, 1·4)						4·7 (3·4, 6·0) 1·7 (1·4, 2·0)					
	78·4	74·8, 82·0	63·4	58·4, 68·4	70·3	65·4, 75·3	81·1	77·3, 85·0	64·6	59·4, 69·9	68·0	62·8, 73·3
Adequacy	89·5 91·7	88·3, 90·7 90·5, 92·9	76·2 79·7	74·0, 78·4 77·2, 82·2	80·6 82·9	78·8, 82·4 80·8, 85·0	88·1 90·1	87·1, 89·1 88·5, 91·6	73·9 78·8	71·9, 75·9 76·7, 81·0	76·0 80·3	74·3, 77·8 78·7, 81·9

CI, Confidence interval; EAR, estimated average requirement; NAR, nutrient adequacy ratio; PA-P, probability of adequacy calculated using the probability method; PA-U, probability of adequacy calculated using usual intake. Descriptions are in online Supplementary S.1.

* Analysis done using complex survey design, weighted analyses.

† Ca: (PA-P as in Foote *et al.*^(4)^, PA-U as in Foote *et al.*^(4)^ and Arimond *et al.*^(6,23)^ (online Supplementary S.4).

‡ Fe and Zn – high bioavailability, use probability of adequacy as described in Gibson and Ferguson^(39)^ for Methods 2 and 3, but use back-transformed intakes for Method 3 (online Supplementary S.2, S.5 and S.6).

**Figure 2.**
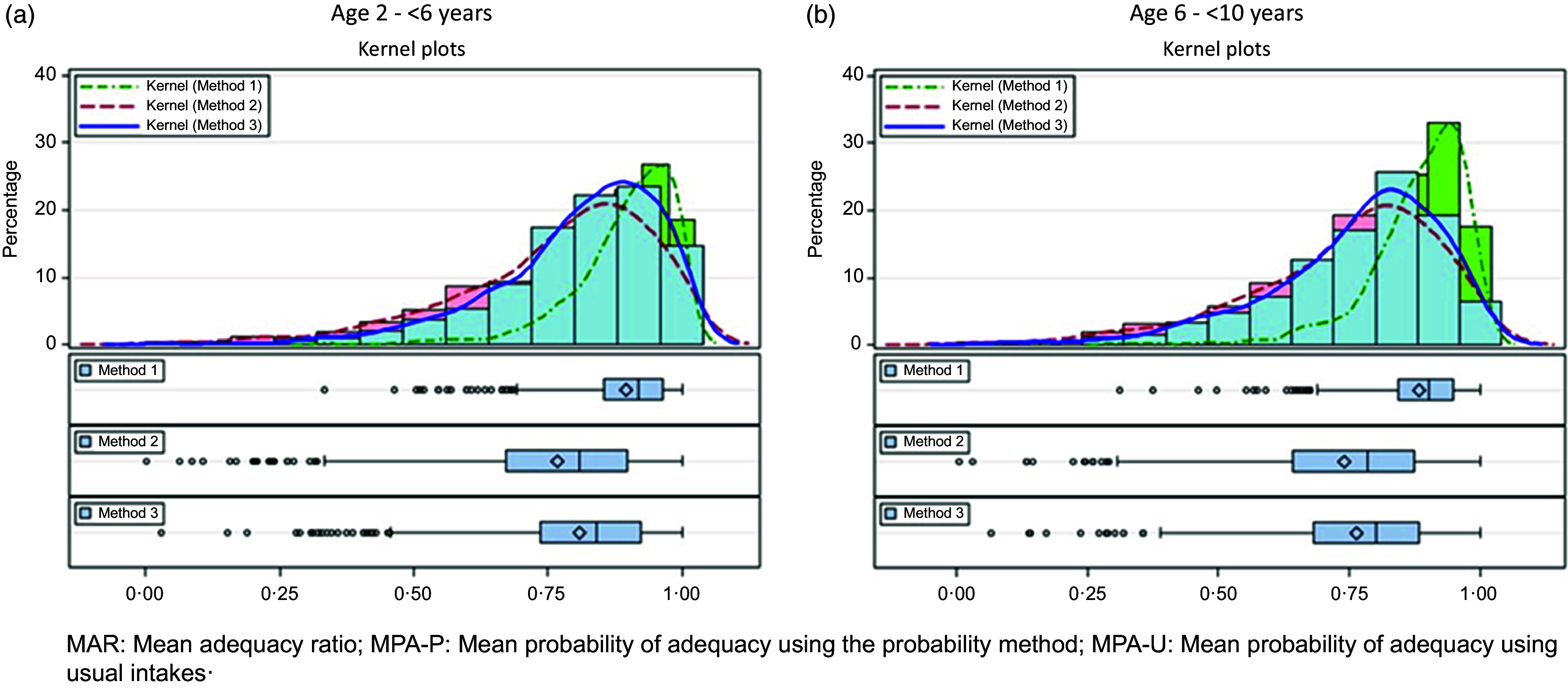
Comparison of the histograms with associated kernel distributions as well as schematic box plots of MAR (Method 1), MPA-P (Method 2) and MPA-U (Method 3), by age group. (a) Comparisons of the histograms for 2–<6-year-olds. (b) Comparisons of the histograms for 6–<10-year-olds.

Figure 2 shows the distributions, by age group, of the mean adequacy measures calculated using the three methods. The normal distribution and kernel distribution, which is a non-parametric representation of the probability density function of a random variable^(48)^, are superimposed on the histograms of the mean adequacy measures calculated using the three methods. The values of the mean adequacy measures range between 0 and 1. The shape of the distributions of the 15 NAR is not normal, they are skewed to the left and truncated at 1, resulting in similar shapes for the MAR. The distribution of MPA-P calculated using Method 2 was also skewed to the left and truncated at 1. A suitable Box-Cox transformation could not be performed to transform the distributions of MAR and MPA-P to normality. The distribution of MPA-U calculated using Method 3 is the closest to normal and was transformed using a Box-Cox transformation to a normal distribution, resulting in a fourth measure, MPA-U-BC.

Figure 2(a) and (b) also shows that the kernel distributions for MPA-P and MPA-U are almost similar for children 2 to <6 years and 6 to <10 years, respectively. The schematic boxplots clearly indicate the higher mean and median values for MAR followed by MPA-U, with MPA-P producing the lowest values.

The associations (Spearman correlation coefficient) between the mean adequacy values calculated using the three methods and the transformed adequacy values (MPA-U-BC), and age (in months), the DD scores, as well as the total energy intake for the dataset for 2 to <10-year-old children are presented in Table 3. Additionally, results of a multiple linear regression analysis with the mean adequacy values as dependent variables and the age, DD scores and total energy intake as independent variables are shown in Table 3. Age (in months) has a significant negative relationship, and the DD scores as well as total energy intake have significant positive relationships with the respective mean adequacy values.

**Table 3.** Spearman correlation and multiple regression analysis with mean adequacy as dependent variable, and selected independent variables, *n* 1170

	MAR (Method 1)		MPA-P (Method 2)		MPA-U (Method 3)		MPA-U-BC (Method 3)	
	Multiple Regression	Spearman Correlation	Multiple Regression	Spearman Correlation	Multiple Regression	Spearman Correlation	Multiple Regression	Spearman Correlation
Adj R^2^	0·449		0·466		0·439		0·462	
Age in months	–0·001***	–0·174^†††^	–0·002***	–0·158^†††^	–0·002***	–0·256^†††^	–0·002***	–0·256^†††^
DD scores	0·029***	0·447^†††^	0·047***	0·408^†††^	0·040***	0·388^†††^	0·031***	0·388^†††^
Total kJ	<0·001***	0·546^†††^	<0·001***	0·585^†††^	<0·001***	0·500^†††^	<0·001***	0·500^†††^

Significant relationship with mean adequacy variable, multiple linear regression analysis, ***P < 0.001.

Significant Spearman correlation coefficient, ^&&&^*P* < 0.001.

MAR, mean adequacy ratio; MPA-P, mean probability of adequacy using the probability method; MPA-U, mean probability method using usual intakes; MPA-U-BC, Box-Cox transformed values of mean probability of adequacy using usual intakes; AdR^2^, adjusted R^2^; DD, dietary diversity.

The evaluation per age group of the AUC for each MPA-U threshold level, and the performance of possible MDD indicators in classifying intakes as adequate, per threshold value, are presented in Tables 4 and 5. Similar tables for MAR and MPA-P are presented in the online supplementary material (Supplementary Tables S.7.1–S.7.4).

**Table 4.** Evaluation of minimum dietary diversity (MDD) indicators for different mean probability of adequacy using usual intakes (MPA-U) thresholds for data from the dataset for 2–<6-year-old children

		MPA-U thresholds‡ for Method 3: age 2–<6 years (*n* 691)			
		≥0·5	≥0·6||	≥0·7	≥0·8
*n* (%) (≥threshold or prevalence of adequate intake)		657 (95·1 %)	622 (90·0 %)	551 (79·7 %)	418 (60·5 %)
Logistic regression: Odds ratio (95 % CI)		3·05*** (2·03, 4·59)	3·11*** (2·28, 4·24)	2·27*** (1·83, 2·81)	2·04*** (1·72, 2·42)
AUC (95 % CI)		0·760^†††^ (0·680, 0·841)	0·763^†††^ (0·709, 0·817)	0·700^†††^ (0·654, 0·746)	0·683^†††^ (0·644, 0·721)
MDD = 3	Sensitivity	0·846	0·862	0·880	0·913
	Specificity	0·529	0·478	0·379	0·304
	LR+ and post probability of TH	1·80 (0·64)	1·65 (0·71)	1·42 (0·77)	1·31 (0·84)
	Youden index	**0·376**	0·340	0·259	0·217
	Euclidean distance	**0·495**	0·540	0·632	0·701
	Accuracy	**0·831**	**0·823**	**0·779**	**0·673**
	Kappa	**0·156**	**0·268**	**0·289**	**0·273**
MDD = 4§	Sensitivity	0·508	0·529	0·546	0·586
	Specificity	0·853	0·855	0·729	0·656
	Youden index	0·361	**0·384**	**0·275**	**0·242**
	Euclidean distance	0·656	**0·493**	**0·529**	**0·538**
	LR+ and post probability of TH	3·46 (0·78)	3·65 (0·85)	2·01 (0·82)	1·70 (0·87)
	Accuracy	0·525	0·561	0·583	0·614
	Kappa	0·067	0·159	0·224	0·257

LR+, positive likelihood ratio test; TH, threshold; ROC, receiver operating characteristic; AUC, area under the ROC curve; MDD, minimum dietary diversity; MPA-U, mean probability of adequacy using usual intakes.

Significant odds ratio, **P < 0.01, ***P < 0.001; Wald Chi square test for ROC contrast, ^††^*P* < 0.001, ^†††^*P* < 0.001.

‡ Threshold levels <0.5 were excluded.

§ Select the MDD indicator for each threshold using the maximum Youden index.

|| The best threshold is 0.6, using the maximum AUC over all thresholds.

**Table 5.** Evaluation of minimum dietary diversity (MDD) indicators for different mean probability of adequacy using usual intakes (MPA-U) thresholds for data from the dataset for 6–<10-year-old children

		MPA-U thresholds‡ for Method 3: age 6–<10 years (*n* 479)			
		≥0·5||	≥0·6	≥0·7	≥0·8
*n* (%) (≥threshold or prevalence of adequate intake)		441 (92·1 %)	409 (85·4 %)	348 (72·7 %)	241 (50·3 %)
Logistic regression: Odds ratio (95 % CI)		2·41*** (1·63, 3·57)	2·04*** (1·54, 2·71)	1·88*** (1·51, 2·33)	2·09*** (1·72, 2·55)
AUC (95 % CI)		0·713^†††^ (0·633, 0·792)	0·683^†††^ (0·619, 0·747)	0·670^†††^ (0·619, 0·721)	0·700^†††^ (0·656, 0·745)
MDD = 3§	Sensitivity	0·841	0·853	0·868	0·909
	Specificity	0·447	0·386	0·313	0·273
	Youden index	**0·289**	0·239	0·181	0·182
	Euclidean distance	0·575	0·631	0·700	0·733
	LR+ and post probability of TH	1·52 (0·60)	1·39 (0·68)	1·26 (0·75)	1·25 (0·83)
	Accuracy	**0·810**	**0·785**	**0·716**	**0·716**
	Kappa	**0·165**	**0·208**	0·206	0·213
MDD = 4	Sensitivity	0·499	0·513	0·549	0·635
	Specificity	0·789	0·743	0·718	0·685
	Youden index	0·288	**0·256**	**0·266**	**0·320**
	Euclidean distance	**0·544**	**0·550**	**0·532**	**0·482**
	LR+ and post probability of TH	2·37 (0·70)	2·00 (0·75)	1·94 (0·82)	2·01 (0·89)
	Accuracy	0·522	0·547	0·595	0·595
	Kappa	0·075	0·130	**0·265**	**0·365**

LR+, positive likelihood ratio test; TH, threshold; ROC, receiver operating characteristic; AUC, area under the ROC curve; MDD, minimum dietary diversity; MPA-U, mean probability of adequacy using usual intakes.

Significant odds ratio, **P < 0.01, ***P < 0.001; Wald *χ*^2^ test for ROC contrast, ^&&^*P* < 0.001, ^&&&^*P* < 0.001.

‡ Threshold levels <0.5 were excluded.

§ Select the MDD indicator for each threshold using the maximum Youden index (J).

|| The best threshold is 0.5 using the maximum AUC over all thresholds.

Comparisons of the AUC values of the different threshold levels of MPA-U ≥ 0·5, ≥0·6, ≥0·7 and ≥0·8, respectively, are shown in Table 4. The best adequacy threshold value for MPA-U is ≥0·6 for 2 to <6-year-old children, considering the AUC value (0·763), but the specificity is below 0·5 for an MDD indicator of 3 and the accuracy level is below 0·6 for an MDD indicator of 4. For MPA-U ≥ 0·5, with an associated AUC value of 0·760, sensitivity and specificity, and both the accuracy levels and Kappa statistic validate the conclusion that an MDD indicator of 3 will be the best associated cut-off point. As soon as the MDD indicator changes to 4, the sensitivity decreases and specificity increases, therefore decreasing the ability to identify subjects with adequate intake. The post-test probability of the associated likelihood ratio of 1·8 (Table 4, MAR ≥ 0·5, MDD indicator of 3) implies that an MDD indicator of 3 is a good choice for a cut-off value, as it could raise the threshold value of 0·5 to 0·64 (by 14 %). Although the post-test probability associated with an MDD indicator of 4 could raise the threshold value of 0·5 to 0·78 (28 %), the associated accuracy level is too low. Both the accuracy level (0·831) and the Kappa statistic (0·158) indicate an MDD indicator of 3 rather than an MDD indicator of 4, if MPA-U ≥ 0·5, confirming the conclusion made following the interpretation of the Youden index and Euclidean distance. Although the Kappa statistic indicates a slight agreement between the MDD indicator level of 3 and the threshold level of 0·5, it is higher for the MDD indicator of 3 than 4.

The maximum AUC is associated with a threshold of 0·5 for children 6 to <10 years, but different MDD indicator values will be chosen when considering the Youden index and the Euclidean distance. The accuracy level and Kappa statistic favour an MDD indicator of 3. In this case, specificity is below 0·5 (Table 5).

Similar interpretations can be made for Tables S.7.1–S.7.4, representing MAR and MPA-P.

**Figure 3.**
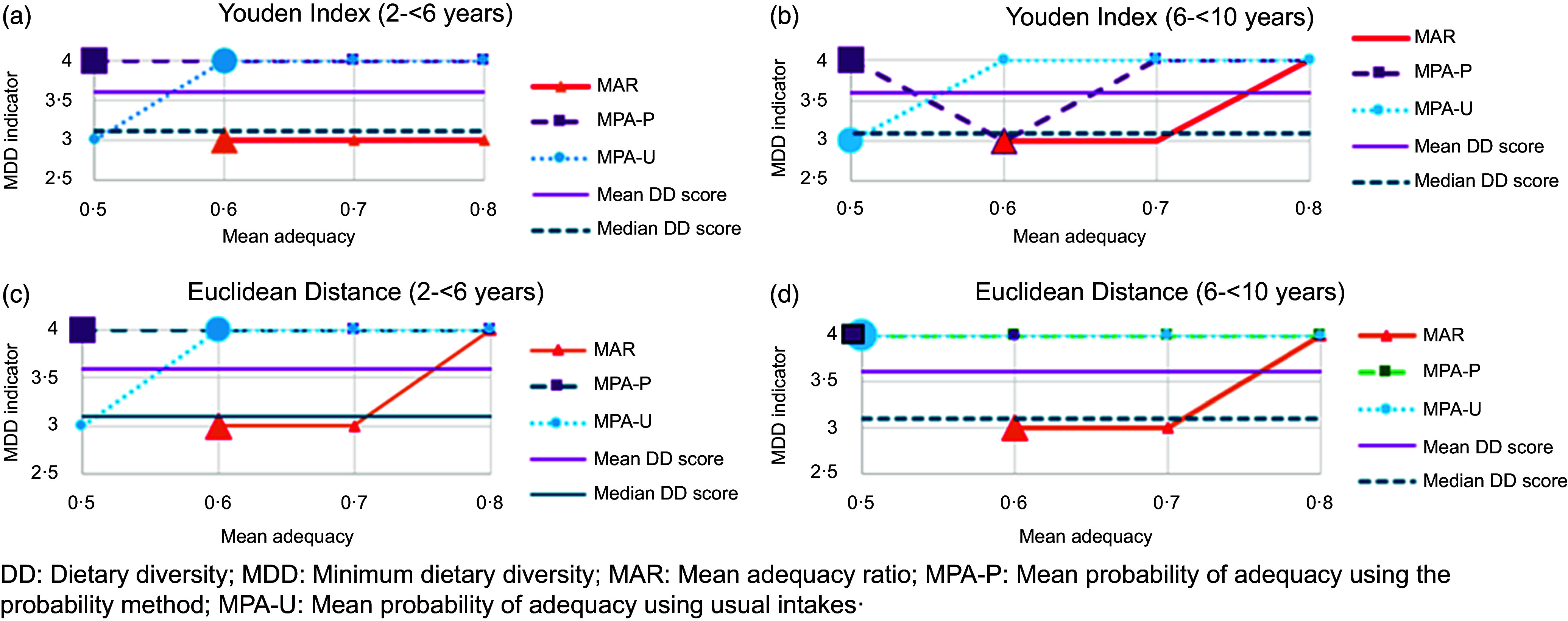
Comparison, by age group, of the position of the minimum dietary diversity (MDD) indicator as calculated using the Youden index and Euclidean distance, respectively, relative to the mean and median of the dietary diversity scores, for different mean adequacy thresholds. The larger dots show the position of the best nutrient adequacy threshold values corresponding to the maximum AUC. (a) The position of the MDD indicator calculated using the Youden index, 2–<6-year-old children. (b) The position of the MDD indicator calculated using the Youden index, 6–<10-year-old children. (c) The position of the MDD indicator calculated using the Euclidean distance, 2–<6-year-old children. (d) The position of the MDD indicator calculated using the Euclidean distance, 6–<10-year-old children.

Figure 3(a)–(d) provides further perspectives on the results presented in Tables 4 and 5 and Tables S.7.1–S.7.4. These figures compare, by age group, the position of the MDD indicator as calculated using the Youden index and Euclidean distance, respectively, relative to the mean of 3·6 and the median of 3·1 of the DD scores, for different mean adequacy thresholds. The larger dots show the best threshold values calculated for the three methods separately using the AUC values. The MDD indicators are either 3 or 4, therefore the integers just above or below the median (and the mean in this case) of the DD scores. Lower sensitivity and higher specificity values are associated with an MDD indicator of 4, which is the first integer above the median DD score. The opposite is true for an MDD indicator of 3. The selected MDD indicator is 3 for both age groups, considering both the Youden index and the Euclidean distance, using MAR (adequacy threshold is 0·6). When using MPA-P, the selected MDD indicator is 4 for both age groups (adequacy threshold is 0·5). Using MPA-U, considering usual intakes, the MDD indicator is 4 for 2 to <6 years (adequacy threshold is 0·6 for 2 to <6 years) and varies between 3 (Youden index) and 4 (Euclidean distance) with an adequacy threshold of 0·5 for 6 to <10 years.

**Figure 4.**
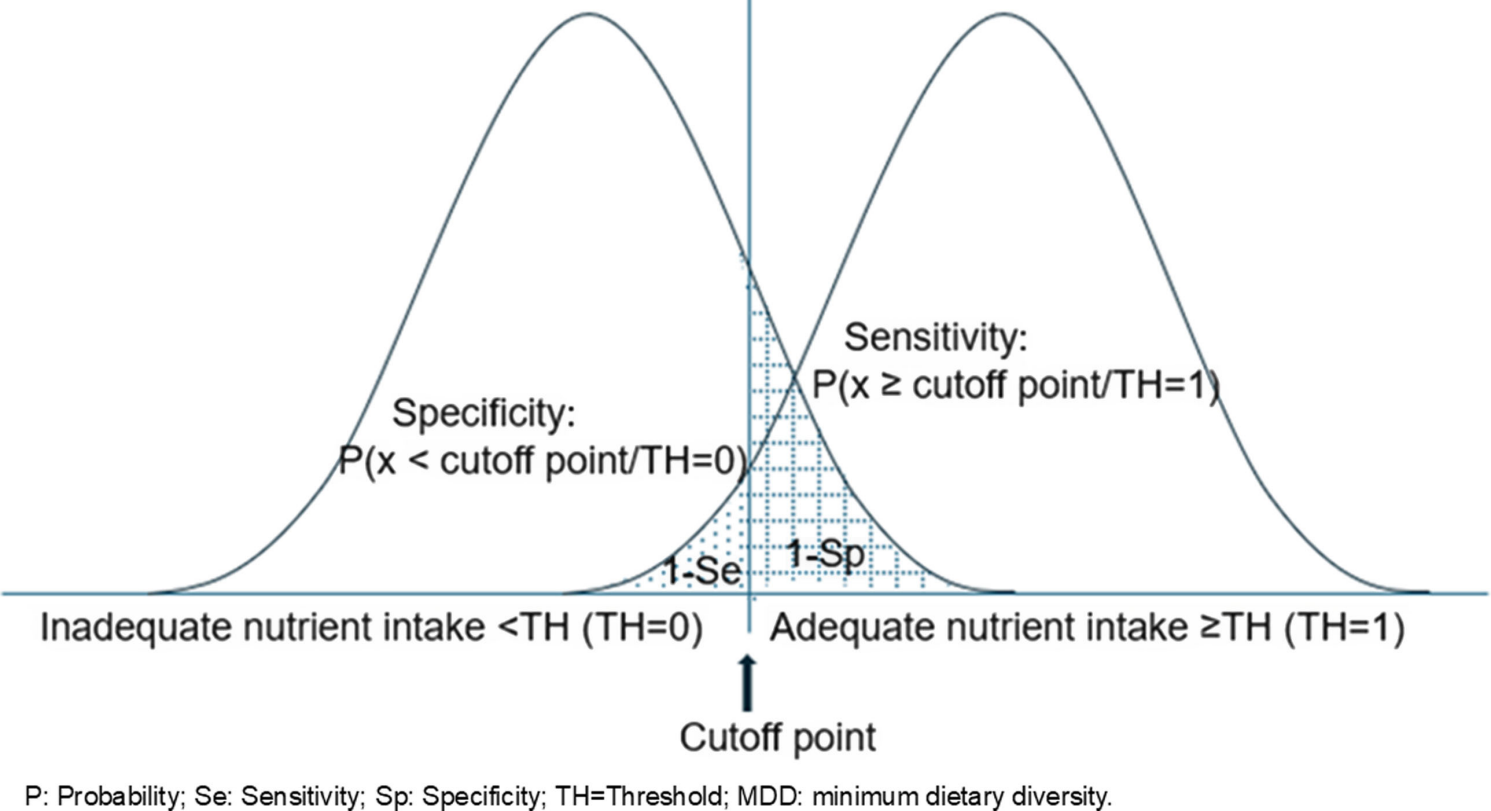
Graphical illustration of two hypothetical distributions for subjects with adequate (≥threshold) and inadequate (<threshold) intake, respectively. The vertical line indicates the position of the hypothetical MDD indicator.

A closer inspection of the calculation of an MDD indicator shows that the groups with adequate and inadequate intake overlap. Figure 4 shows two hypothetical distributions for subjects with adequate (≥ adequacy threshold) and inadequate (<adequacy threshold) intake, respectively. The vertical line indicates the hypothetical position of the MDD indicator. In this situation, for a given MDD indicator subjects with adequate intake who have a DD score < MDD will be classified incorrectly (false negative or 1—sensitivity). If the MDD indicator is decreased to increase sensitivity of the test, the number of false positives (1-specificity) increases, decreasing specificity. Therefore, lower MDD indicators correspond to lower specificity values and higher sensitivity and vice versa.

**Figure 5.**
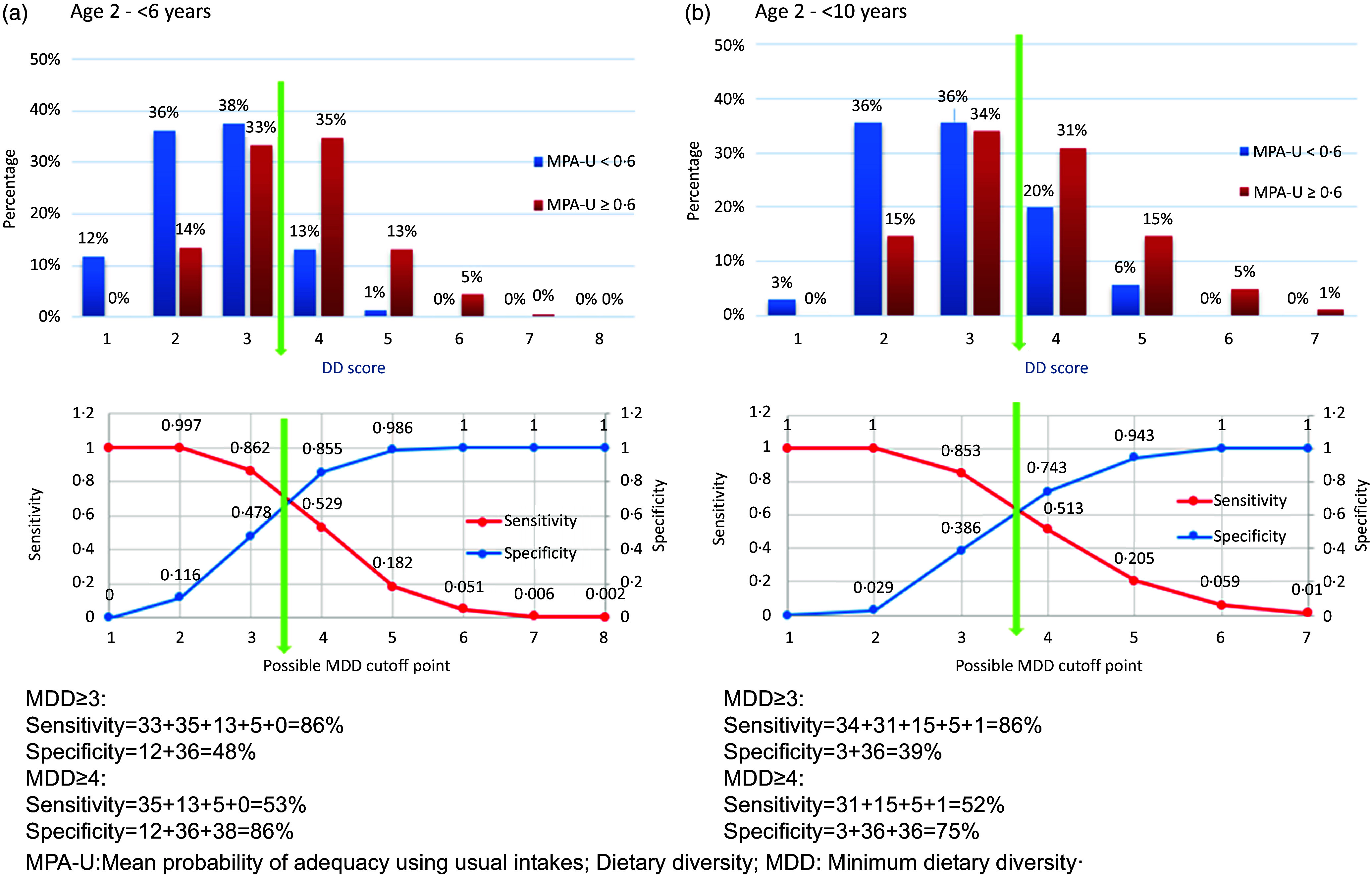
Demonstration of the calculation of sensitivity and specificity for identification of a possible minimum dietary diversity (MDD) indicator to the left and right of the median of the dietary diversity (DD) scores, using a mean probability of adequacy for usual intakes (MPA-U) threshold of 0·6 (Method 3). (a) Calculation of sensitivity and specificity for 2–<6-year-old children. (b) Calculation of sensitivity and specificity for 6–<10-year-old children.

Each figure in Fig. 5(a) and (b) (Method 3, using MPA-U), and online Supplementary Figs. S.8.1(a) and (b) (Method 1, using MAR) and S.8.2(a) and (b) (Method 2, using MPA-P) demonstrates, by age group, two frequency distributions of adequate intake, below and above an example threshold value of 0·6, respectively, per DD score. The percentage of sensitivity and specificity for the given threshold value per figure, by MDD indicator (cut-off points), are displayed in the corresponding bottom figure. The position of the median of the DD scores is also indicated. In these figures, sensitivity and specificity are calculated for the MDD to the left and the right of the median of the DD scores. In each case, the MDD indicator to the left of the median results in higher sensitivity and lower specificity values, and the MDD indicator to the right of the median results in higher specificity and lower sensitivity values.

The count of the number of cases greater or equal, or less than each possible MDD indicator is displayed in Table 6. The resulting MDD indicator is selected amongst either the highest DD score for which the number of subjects with DD score ≥ MDD indicator is greater than L_50_ (in this case MDD indicator ≥3) or the smallest DD score for which the number of subjects with DD score ≥ MDD indicator is less than L_50_ (in this case MDD indicator ≥4). In the first case, sensitivity is usually (most of the times) higher than specificity, and vice versa for the second case. In Table 6, the resulting change from higher sensitivity and lower specificity to a lower sensitivity and higher specificity takes place between the MDD indicators of 3 and 4, which are the integers just below and above the median value. Table 6 also shows the corresponding sensitivity and specificity values for each possible MDD indicator, and the associated Youden index. The maximum Youden index is obtained for that MDD indicator corresponding to the integer smaller than the median of the DD score (MDD indicator of 3), in this case. It is also possible that the Youden index can obtain a maximum at the first MDD indicator greater than the median of the DD score.

**Table 6.** Detailed explanation of the role of the position of the median in selecting the minimum dietary diversity (MDD) indicator, children aged 2–<6 years, and a mean adequacy ratio (MAR) threshold of 0·6, as an example

Possible MDD	Calculations for this possible MDD indicator			Remarks* (L_50_ = 345·5)	Performance criteria				
	Adequate intake	Inadequate intake	Total in MDD group		Sensitivity: a/(a + b)	Specificity: d/(c + d)	Youden	Eucl. Distance	Acc
≥2	672 (a)	9 (c)	681 (a + c)		0·99	0·25	0·24	0·75	0·98
<2	7 (b)	3 (d)	10 (b + d)						
Total in adequacy group	679 (a + b)	12 (c + d)	691 (a + b + c + d)						
≥3	568	4	572	The number of subjects with a MDD ≥ 3 is more than the position of the median (572 > 345·5).	0·84	0·67	**0·50**	**0·37**	**0·83**
<3	111	8	119						
Total in adequacy group	679	12	691						
≥4	338	1	339	The number of subjects with MDD ≥ 4 is less than the position of the median (339 < 345·5).	0·50	0·92	0·42	0·51	0·51
<4	341	11	352						
Total in adequacy group	679	12	691						
≥5	114	0	114		0·17	1·00	0·17	0·83	0·18
<5	565	12	577						
Total in adequacy group	679	12	691						

(a)–(d): Symbols and formulas used to explain the calculations of sensitivity, specificity and the value of the Youden index.

Eucl., Euclidean; Acc, accuracy; MDD, minimum dietary diversity.

* L_50_ = 345.5. The position of the median of 3.1 is between observations 345 and 346, and the value of the median lies between 3 and 4. The resulting change from higher sensitivity and lower specificity to a lower sensitivity and higher specificity takes place between the MDD of 3 and 4.

## Discussion

In this study, we set out to illustrate a process approach for the calculation of MDD indicators to interpret DD scores and to validate the MDD indicators as a proxy for adequate intake using an existing dataset of 2 to younger than 10-year-old South African children. For these purposes, we used nine food groups to derive the DD scores instead of the ten food groups as specified by the FAO^(8)^ for women by combining the pulses and nuts and seeds groups. Menber *et al.* ^(26)^ noted that variations in dietary practices in specific countries or regions should be noticed when defining food groups for use in the calculation of MMD indicators. Disparities among different countries lead to inconsistencies in the findings of studies concerning the actual utilisation of MDD-W indicators as proxy indicators of micronutrient adequacy^(26)^.

Three methods were used to calculate micronutrient adequacy, based on the older methods described by Hatløy *et al.* ^(1)^ and Foot *et al.* ^(4)^ (MAR and MPA-P), which are still commonly used when only a single 24-h recall is available^(17,18,25–28)^ and the more recent methods (MPA-U) described by Martin-Prevel *et al.* ^(42)^ when usual dietary intake data is available. The results of the older methods are still meaningful in predicting adequacy for individuals in the absence of more than one intake assessment, but do not account for within-person variation. Kennedy *et al.* ^(16)^ noted that not accounting for the within-person variation could affect the mean adequacy values as well as perhaps the selected MDD indicators. Our results show that the average of the MAR (Method 1) values (89·5 and 88·1 %) are generally the highest (only one 24-h recall), followed by the average of the MPA-U values (80·6 and 76·0 %) and lowest are the average of the MPA-P (Method 2) values (76·2 and 73·9 %), for both age groups. The position of the MDD indicator tends to favour the integer above the median of the DD scores when using the probability methods, whereas the position of the MDD indicator tends to favour the integer below the median when using the MAR. Although we agree partly with Kennedy *et al*.^(16)^, the results from the two probability methods generally agree. However, not adjusting for within-person variance could affect the mean adequacy values as well as further conclusions regarding mean adequacy in populations and associations with additional study outcome variables. Hanley-Cook *et al.* ^(25)^ also noted that healthy diet metrics based on a single 24-h recall lack precision.

Following a process approach, an MDD indicator of three or four food groups out of the nine SA-Child food groups was statistically supported for 2 to younger than 6-year-old and 6 to younger than 10-year-old children, irrespective of the method used to calculate dietary adequacy, with higher sensitivity and lower specificity corresponding to an MDD indicator of three, and higher specificity corresponding to an MDD indicator of four. This is in contrast with the MDD indicator of five out of ten groups suggested by the FAO for adult women^(8)^ and the MDD indicator of five out of eight groups^(7)^ suggested by the WHO and UNICEF for children 6 to <24 months old. When considering further literature on MDD indicators in all age groups, it is evident that these indicators vary from study to study^(26)^, depending on the population under investigation. Even within the same population different MDD indicators according to children’s age and place of residence have been proposed^(49)^.

The statistical process for calculation and validation of the MDD indicators involved identification of threshold levels of adequate intake and associated MDD indicators for each of the three methods. For identification of the ‘best’ MDD indicator, we found that the point of intersection of sensitivity and specificity curves, as indicated in Fig. 5 and online Supplementary Figs. S.8.1 and S.8.2, identifies micronutrient adequacy and inadequacy. This technique has been commonly used in similar studies^(1,5,16,27,43,49,50)^. It is not always clear whether an MDD indicator to the left or the right of the intersection of the two lines should be used, i.e. whether sensitivity or specificity should be prioritised^(29)^. Performance criteria, such as the Youden index and the Euclidean distance, are useful in this regard^(43,51)^, as is also evident from our results.

The mean DD score was 3·6, and median was 3·1 for both children 2 to <6 years and 6 to <10 years. Evidence presented in the results suggest that the MDD indicator increases from the integer below the median (and mean) to the integer above the median (and mean) of the DD scores as the adequacy thresholds increase. Using the suggested performance criteria outlined above, the appropriate MDD indicator is therefore either three or four for this dataset, giving preference to sensitivity in the case of three food groups and specificity in the case of four food groups. An important finding in this study, irrespective of the method of calculating adequate intake or the threshold level of adequate intake used, is that performance criteria such as the Youden index and the Euclidean distance suggested that the MDD indicator will be the integer either above or below the median (and mean) of the DD scores.

Inspection of the results of other studies (online Supplementary Tables S.9.1 and S.9.2) reflects the same outcome, irrespective of the performance criteria applied. Table S.9.1 demonstrates trends similar to those depicted in Figs. 3 and 5 in the present study. When inadequate intake was evaluated (Table S.9.1), the MDD indicator selected was the first integer greater than the mean of the DD score^(1,5,12,16,49,50,52)^, in the absence of known median values. In these studies, sensitivity measured the ability to identify inadequate intake, and sensitivity was higher than specificity. However, the strategies used to select the MDD indicator differed. Schutte *et al.* ^(12)^ and Hatløy *et al.* ^(1)^ have preference to sensitivity, Steyn *et al.* ^(5)^, Kennedy *et al.* ^(16)^, Zhao *et al.* ^(49)^ and Torrico *et al.* ^(52)^ selected the MDD indicator by considering a balance between sensitivity and specificity and Steyn *et al.* ^(50)^ considered a higher sensitivity, but lower misclassification.

Further evidence in this regard comes from results from four countries, Burkina Faso, Mali, Mozambique and the Philippines, in the study by Arimond *et al.* ^(23)^ where the emphasis is on adequacy, and the MDD indicator was selected as the first integer greater than the mean DD score (online Supplementary Table S.9.2). To select the MDD indicator, they considered criteria such as a balance between sensitivity and specificity (preferably both ≥ 60 percent; still considered if one of the two only was ≥ 50 percent) and the rate of misclassification (preferably ≤ 30 percent; still considered if ≤ 40 percent). In these studies^(23)^, sensitivity measured the ability to identify adequate intake but for most of these countries, specificity was favoured. This means that the researchers chose to identify participants with low MPA-U and accept that some with a higher MPA-U value will be classified incorrectly. Bangladesh, the fifth country in the study by Arimond *et al.* ^(23)^, was different as the MDD indicator was identified as 5, although the mean of the DD score was 3·6. Their conclusion was that the diets of women at this site would be the most monotonous among the five sites examined, which is the reason for selecting an MDD with the higher specificity (84·6 %), as opposed to an MDD indicator with a higher sensitivity (MDD = 4 at sensitivity = 83·1 %). If the Youden index is applied on the Bangladesh data, the maximum would have been at an MDD indicator of 4, as demonstrated in online Supplementary Table S.9.3. Other studies^(29–31)^ investigating adequate intake (online Supplementary Table S.9.2) also considered a balance of sensitivity and specificity and obtained MDD indicators just below or above the mean of the DD scores. Furthermore, Monge-Rojas^(32)^ incorporated the Youden index in their decision.

We conducted further analysis on the Arimond *et al.* ^(23)^ datasets to illustrate the validity of our proposal that the MDD indicator lies either above or below the median (and possibly the mean) of the DD score (online Supplementary Tables S.9.2 and S.9.3). Results showed that it is possible to establish the position of the median (L_50_) using the sample size and then to count the number of cases greater or equal or less than the MDD indicator. The corresponding sensitivity and specificity values for those cut-off points were provided,^(23)^ and we calculated the maximum Youden index (online Supplementary Table S.9.3). We established that this Youden index value corresponded to the MDD indicator selected by the authors^(23)^. Also, the desired MDD indicator was either below or above the mean of the DD score (in the absence of the median) for each sample, supporting our finding in this regard.

Kennedy^(16)^ argued that the decisions regarding the most appropriate MAR/MPA to be used to define the MDD indicator, as well as whether sensitivity or specificity (or both) is more important, will eventually depend on the intended use of the MDD indicator. For example, if the goal is to identify children with adequate micronutrient intake, one would aim to maximise sensitivity (in the case of assessing adequacy) therefore reducing specificity and thus including more children who are truly at risk in the target group. Arimond *et al.* ^(23)^ noted that, although it is reasonable to aim for a balance between sensitivity and specificity, specificity should be favoured when trade-offs must be made. This will identify all those with inadequate nutrient intake and may include some children with adequate intake incorrectly classified as having inadequate intake. Using performance criteria such as the Youden Index or the Euclidean distance simplifies the decision-making process.

Finally, when a given MDD indicator has been established based on statistical indicators, which is three or four for our existing dataset of 2 to <10-year-old children, closer inspection of the appropriateness thereof is essential, especially as the MDD indicators are lower than the proposed MDD-W cut-off point of 5 (for both adult women^(8)^ and for children 6 to <24 months^(7)^). The FAO^(53)^ stated that with an MDD indicator of at least four food groups the previous day, a child in a certain population would have a high likelihood of consuming at least one animal source food and at least one fruit or vegetable in addition to a staple food (WHO/UNICEF 2010 guidelines). The lower MDD indicator we derived for our study sample may be the result of a diet high in starchy staples (which supplies 40·6 % of total energy intake) and meat (12·9 % of total energy intake). The fortification of staple starches (consumed by 100 % of the sample), namely bread and maize flour as mentioned earlier, may have contributed to high nutrition adequacy ratios and probability of adequacy of these nutrients^(24)^. However, inadequate intake of nutrients that are not part of the fortification mix such as Ca and vitamin C may be concealed by an MDD indicator of 3. This possibility is supported by the finding that intake for all children from the dairy group was 115 g (recommendation = 500 g for children 2–3 years and 625 g for children 4–8 years^(54)^) and 97 g for all fruit and vegetables combined (recommendation = 320 g for children in the pre-school age group and for school children at least 400 g every day^(55)^). It might be prudent to follow the reasoning by Arimond *et al.* ^(23)^ to increase the desired MDD indicator to four food groups, irrespective of what the performance indicators such as the Youden Index suggest. A further point when evaluating a statistically supported MDD indicator is that portion size consumed is not considered, which may also result in concealing of inadequate consumption. Within the South African context this notion is supported by the work of Faber and colleagues^(56)^ who noted that although fortified staples are frequently consumed by infants and toddlers (6–24 months), the micronutrient density of the complementary diet was inadequate for several key nutrients such as Ca.

### Conclusions

In this study, we illustrated a systematic process for the establishment of an appropriate MDD indicator for the interpretation of DD scores calculated from nine food groups using an existing dataset of 2 to younger than 10-year-old children. We conclude that inspection of micronutrient adequacy using different methods, including MAR, MPA and MPA-U, depending on the available datasets, and inspection and validation of adequacy threshold levels and candidate MDD indicators using several performance criteria, including sensitivity, specificity, the Youden index, the Euclidean distance, the likelihood ratio test, accuracy measures and Cohen’s Kappa, resulted in clear identification of statistically supported MMD indicators for the children in the dataset. The resulting MDD indicator varied between three out of nine food groups favouring the identification of children with adequate intake, and four out of nine food groups favouring the identification of children with inadequate intake, depending on the method used. These MDD indicators were the integer above and below the mean and median DD scores of 3·6 and 3·1, respectively. We therefore further conclude that a simplified method or ‘rule of thumb’ for determination of an MDD indicator is to establish the integer values below (sensitivity) and above (specificity) the median of the DD score. Irrespective of whether the MDD indicator was derived using the full systematic process, we described or via the simplified method, the appropriateness thereof within the country or community specific context should be considered in the setting of the final MMD indicator for application in interpretation of DD scores.

## Supporting information

Nel et al. supplementary material Nel et al. supplementary material

## Data Availability

The data presented in this study are available on request from the corresponding author pending ethical approval from the Faculty of Health Sciences Human Research Ethics Committee, University of Cape Town.

## Supplementary material

For supplementary material/s referred to in this article, please visit https://doi.org/10.1017/S0007114525000807
