# Supplementary material for: Illustration of a process for the calculation and validation of minimum dietary diversity indicators using an existing dataset of 2 to younger than 10-year-old children
Source: Br J Nutr. 2025 Apr 10;133(9):1254–68. doi: 10.1017/S0007114525000807 (PMC12229985; doi:10.1017/S0007114525000807)
Supplement: Nel et al. supplementary material [file S0007114525000807sup001.docx]

**Supplementary files**

**Supplementary S.1**

**Description for Supplementary Table S.1**

*Fixed cutoff points methods:* The nutrient adequacy ratio (NAR) and the mean adequacy ratio (MAR) are widely used measures of dietary quality which reflect nutrient adequacy for individuals. This concept was first developed by Madden and Yoder^(1)^. The NAR is the ratio of the respondent’s actual intake of a nutrient to a dietary reference intake for age and sex categories for that nutrient truncated at one^(1,2,3,4)^. Initially, reference intakes that were used were the full recommended dietary allowance (RDA) or for example two-thirds of the RDA^(5,6,7)^. These techniques were also referred to as fixed cutoff points methods, as the estimates of the prevalence of inadequate intake are based on a fixed proportion of the RDA^(7)^. However, in recent dietary diversity DD research the estimated average requirement (EAR) is more commonly used to calculate NARs^(8 – 11)^. The MAR is calculated as the sum of NARs of the select number of micronutrients truncated at 1.0, divided by the number of nutrients considered. The number of nutrients included in calculating the MAR has varied across studies, but micronutrients typically included are vitamin A, vitamin C, thiamine, riboflavin, niacin, folate, iron and calcium^(12)^.

*Probability approach*: The EAR approach to assess the probability of adequate nutrient intake (PA) for an individual can be applied using data from a single 24-hour recall^(7,13,14)^. These methods consider the likelihood that persons with a particular level of intake would fail to meet their nutrient requirement, by comparing the distributions of the nutrient requirement and nutrient intake^(7,15)^. Foote *et al.*^(13)^ indicated in their calculation of mean probability of adequacy (MPA) for individuals using their probability approach that it is not necessary to adjust intakes for the effect of day-to-day variation when computing the PA for an individual on a single day if the distributions are normalised. However, adjustment would be required to accurately estimate the MPA for usual intakes within a population^(13,15)^. Kennedy *et al.*^(14)^ also flagged the use of a single 24-hour recall as a limitation to evaluate MPA for populations, as it is not possible to correct for within-person variation of intake. This could affect the MPA as well as further conclusions regarding MPA in populations per se, and associations between MPA and additional study outcome variables.

*EAR cut-point methods*: A less parametrized version of the probability approach was developed to estimate the prevalence of nutrient adequacy in a group even when it is difficult to determine the underlying probability model precisely^(15)^. The principle is to estimate prevalence of inadequate intakes as the proportion of the population with usual intakes below the EAR^(12,15,16,17,18,19)^. Usual intakes for a population can be obtained by statistical modelling, which mitigates the effect of within-person variation by incorporating data from additional 24-hour recalls for the full sample or a sub-sample^(7,20,21)^. These methods for calculating PA and MPA are generally referred to as EAR cut-point methods^(15)^.

Supplementary Table S.1: Brief summary of the development of the application of dietary diversity scores

| Author, year and dietary method used | Objective | Milestone | Adequacy ratios | Nutrients | Food groups and dietary diversity score (DDS) | Test for, tests used: | Adequacy threshold, DDS cut-off values |
| --- | --- | --- | --- | --- | --- | --- | --- |
| **Nutrition adequacy ratio (NAR) and mean adequacy ratio (MAR) for individuals** | | | | | | | |
| Madden & Yoder, 1972^(1)^. (Families in poverty) and  Madden et al. 1976^(3)^, 76 subjects aged sixty years or older, 24-hour recall. |  | Define concept of nutrient adequacy. Used Recommended Dietary Allowances (RDAs). |  |  |  |  |  |
| Guthrie & Sheer, 1981^(2)^. Includes 212 university students, 24-hour dietary record. | A simple dietary score can substitute complete dietary analysis to evaluate program effectiveness. | Introduce a food group score based on the Basic Four Food Guide^(22)^ and relate that score to nutrient adequacy. | NAR, ratio of actual intake to the relevant RDA, truncated at 1. MAR average of NARs. | 12 nutrients:  Protein, calcium, magnesium, zinc, vitamin A, ascorbic acid, thiamine, riboflavin, vitamins B6 and B12, and folate. | Used milk and milk products, protein/animal foods, fruits and vegetables, bread and cereals. Also added fat and/or oil. | Correlation analyses used. | A significant relationship exists between MAR and DDS. |
| Krebs-Smith *et al.* 1987^(4)^. Includes 3701 individuals one year and older from the USDA 1977-1978 Nationwide Food Consumption Survey. 3-day records used. | Study the effects of dietary variety on dietary quality of individuals. | Relate the DDS to nutrient adequacy. | NAR, ratio of actual intake to the relevant RDA, truncated at 1. MAR average of NARs. | 11 nutrients: Protein, calcium, iron, magnesium, phosphorus, vitamin A, thiamine, riboflavin, vitamins B6, B12 and C | Used 5 major food groups: dairy, grains, fruits, vegetables and fleshy foods. Used 3-day average of score, which is the number of food groups consumed over the three days. From Better Eating for Better Health^(23)^ | Multiple linear regression | Investigate relationships.  There is a positive relationship between variety of food group intake and MAR. |
| Schuette *et al.* 1996^(24)^, first study to evaluate the usefulness of The Food Guide Pyramid^(25)^ as a quantitative tool for assessing nutritional adequacy. Food records of 2489 college students were included. | Determine whether one serving of the Food Guide Pyramid for food groups can screen as a quantitative tool to measure inadequate nutrient intake. | Introduced sensitivity and specificity as performance measures. | NAR, ratio of actual intake to the relevant RDA, truncated at 1. MAR average of NARs (referred to as MAR-5). | 5 nutrients: calcium, iron, magnesium, vitamin A and vitamin B6 evaluated. | Food Guide Pyramid^(25)^ – 5 groups: cereals, vegetables, fruit, dairy, combination of fleshy foods, pulses, nuts and eggs. | Assessed inadequacy using sensitivity. | A MAR-5 score of 75 or greater was defined as nutritionally adequate. Food scoring system had high sensitivity but moderate to low sensitivity. |
| Hatløy *et al.* 1998^(5)^.  Included 77 children  aged 13-58 months in  South-Eastern Mali,  3-day weighed record used. | Count of food groups can predict nutritional adequacy for individuals. | User Receiver Operating Characteristics (ROC) curves to demonstrate the use of sensitivity and specificity, increased the number of food groups. | NAR, ratio of actual intake to the relevant RDA, truncated at 1. MAR average of NARs. | 11 nutrients: Energy, protein, fat (% of energy), vitamins A & C, thiamine, riboflavin, niacin, folate acid, iron, calcium. | 8 groups: grains, dairy, meat, fish, eggs, fruit, vegetables, and green leaves. | Inadequacy, assess proportions with lower MAR, using high sensitivity, keep specificity as high as possible. ROC curves used. | MAR=0.75 (ideal range from 0.7 to 0.85).  Cut-point DDS=6 for identifying inadequacy. |
| Steyn *et al.* 2006^(6)^: National Food Consumption Survey (NFCS) 1999, weighted sample of 2200 children 1-9 year-old in South Africa. Single 24-hour recall used | Assess whether DDS is a good indicator of nutrient adequacy for individuals. | Use Food & Agricultural Organisation (FAO)^(26)^ food groups, select 50% as a threshold for MAR, RDAs used. | NAR, ratio of actual intake to the relevant RDA, truncated at 1. MAR average of NARs. | 11 nutrients: vitamins A, B6, B12 and C,  niacin, thiamine, riboflavin, folate, calcium, iron and zinc. | 9 food groups:  (Cereals, roots,  and tubers, vitamin-A-rich fruits and vegetables, other  fruit, other vegetables, legumes and nuts, meat,  poultry and fish, fats and oils, dairy, eggs. | Inadequacy, assessed proportions with lower MAR, using high sensitivity, keeping specificity as high as possible. ROC curves used. | If one selects 50% as a threshold for MAR then a  DDS of 4 is most appropriate. It gives a sensitivity of  75% and a specificity of 70%. |
| Oldewage-Theron & Kruger, 2008^(27)^: Sample consists of 170 randomly selected elderly respondents in Sharpeville, South Africa. Two 24-hour recalls calculating MAR and a 7-day food frequency used to obtain DDS.  (Similar studies using Dietary Reference Intakes (DRIs): Oldewage-Theron & Kruger, 2011^(9)^; Islam *et al.* 2023^(10)^, and Zhong *et al.* 2022^(11)^). | Assess whether  DDS give a fairly good assessment of the adequacy  of the diet for individuals | Define sensitivity as ability to identify adequate intake, using Estimated Average Requirements (EARs). | NAR, ratio of actual intake to the relevant EAR/AI, truncated at 1. MAR average of NARs | 14 nutrients: calcium, magnesium, copper, selenium, iodine, vitamins A, B2, B6,  C, D, E, pantothenate, folate, and biotin | 9 food groups (FAO)^(26)^:  (Cereals, roots & tubers, other vegetables, vitamin A rich fruit & vegetables, flesh foods, fats & oils, dairy, other fruit, legumes & nuts, eggs. | Adequacy is assessed, proportions with higher MAR, using high sensitivity (identifying adequate intake), keep specificity as high as possible. ROC curves used. | A cut-off point for DDS  of 6 gives a sensitivity of 90% and specificity of 43%. If the cut-off  point is increased by one point to 7, the sensitivity would increase  to 100% and the specificity would decrease to only 11%. |
| **Probability of adequacy (PA) and mean probability of adequacy (MPA) using a single 24-hr recall for individuals** | | | | | | | |
| National Research Council (NRC), 1986^(7)^: One 24 hour recall used. | Calculate MPA using usual intake, to assess adequate intake for individuals. | Recognise distributions of nutrients, assume normal distribution, introduce PA and MPA. | Calculate standardized intake for each nutrient as z=(observed intake of an individual-Estimated Average Requirement (EAR)/standard deviation (SD), the SDs are calculated using EAR x coefficient of variation (CV), where CVs taken from Institute of Medicine (IOM). Consequently, PA=  PROBNORM(z)  MPA=average PA. | Depending on the study  assumptions are that most nutrients under investigation are approximately normal. | Not applicable. | Prevalence of inadequacy in a population. | Not applicable. |
| Foote *et al.* 2004^(13)^:  Included 4969 men and 4800 women,19 years old who participated in the Continuing Survey of Food Intakes  for Individuals 1994–1996, conducted by USDA, District Columbia, USA. Single 24-hour recalls used. | The aim of this study was to determine whether a commodity-based measure of dietary  variety in this population was associated with the probability of nutrient adequacy. | Apply NRC method, relate DDS^(25)^ to MPA, using adequacy | PA as in NRC^(7)^ method.  PA for calcium and iron were calculated differently. MPA was the average of 15 PAs. | 13 nutrients: (Vitamins A, C, E, B-6, B-12,  thiamine, riboflavin, niacin, folate, phosphorus, magnesium, copper,  and zinc. | 5 food groups from the Food Guide Pyramid: Dairy, fruit vegetables, grains and meat. Restrictions on minimum quantities consumed were considered for inclusion of food in calculating the DDS. | Prevalence of adequate intake in a population. Linear regression models were applied. | One 24-hour recall is adequate to calculate PA. |
| Kennedy *et al.* 2007^(14)^. Included 3164 Filipino children aged 24-71 months. Single 24-hour recalls.  See also Zhao *et al.* (2017)^(28)^, Rani (2010)^(29)^. | Assess whether the DDS is based on a simple count of food groups consumed and DDS using a 10-g minimum intake for each food group are good indicators of adequate micronutrient intake in this population. | Investigate how sensitivity and specificity change as threshold on MPA changes, also used a minimum intake of 10g for including a food group. | PA as in NCR method.  SDs are calculated using EAR x CV, where CVs are taken from the IOM. PA of iron was calculated differently. MPA was the average of 11 PAs. | 11 nutrients: Vitamin A & C, thiamine, riboflavin, niacin, vitamins B6 & B12, folate, iron, absorbed calcium and absorbed zinc. Bioavailability of the micronutrients was considered. | The 9 food groups are  cereals and tubers; meat, poultry and fish; dairy; eggs; pulses and nuts;  vitamin A-rich fruits and vegetables; other fruit; other vegetables; oils  and fats. | (Inadequacy) Sensitivity indicates the  percentage of children truly at risk (low MPA) who are correctly  classified by low DDS. ROC curves, linear regression used. | Using an MPA threshold of 50%, the best DDS cutoff point (where the sensitivity and specificity  curves meet) is 5 food groups. Increasing the MPA threshold to 75% increased the DDS cutoff point to 6 food groups. |
| **Probability of adequacy (PA) and mean probability of adequacy (MPA) using more than one 24-hr recall for usual intake of populations.** | | | | | | | |
| Joseph & Carriquiry (2010)^(16)^: Included 303 nonpregnant,  nonlactating (NPNL) women aged 15–49 years from the original Bangladesh  study sample. Of the 303 women, 92 were interviewed on 2 non-consecutive  occasions and their 24-hour food consumption were recorded on both days. The remaining 211 women were interviewed once and thus  their 24-hour food consumption was observed for 1 day only.  Also applied by Arimond *et al.*  2009^(12)^. | Methods for estimating the prevalence of nutrient adequacy  have been proposed^(12)^ and can be implemented when a  minimum of 2 daily food intake observations are collected for at  least some sample individuals. | Calculate MPA based on usual intake using more than one 24-hour recall.  Use different food group indicators.  Address relationships between MPA and regression models. Conclusions applicable to populations. | Probability of adequate daily nutrient intake for a  woman by comparing her estimated usual intake to the appropriate  distribution of nutrient requirements.  Therefore,  calculate PAs by transforming nutrient intakes, calculate BLUPs, simulate requirement distributions and transform these distributions. Lastly, calculate PA for each nutrient. | 9 nutrients: Vitamin A, vitamin C, thiamine, riboflavin, niacin, vitamin B-6, folate,  calcium, iron, and zinc. | Different food group indicators were investigated using 6, 13 and 21 food groups. The indicators also vary regarding the amount of food (15 g) that must be consumed for the food group to count. | Linear regression | The linear regression model detected significant associations between food group performance indicators and  MPA as well as for variations of these measures, including the best linear unbiased predictor. |
| Arimond *et al.* (2011)^(30)^. A subset of women’s dietary intake data from surveys undertaken by the International Food Policy Research Institute (IFPRI) and collaborators in 1996 were used. The data sets analyzed as part of the Women’s Dietary Diversity Project (WDDP) are from sites in Bangladesh, Burkina Faso, Mali, Mozambique and the Philippines. Two 24-hour recalls, 412 women from the first round and 147 from the second round.  See also Martin-Prevel *et al.* (2015)^(17)^, Arsenault *et al.* (2013)^(18)^, Caswell *et al.* (2018)^(31)^, Diop *et al.* (2020)^(19)^. | To assess the potential of simple indicators of dietary diversity to function as proxy indicators of diet quality. | WDDP/ FAO  Comprehensive study to investigate performance indicators, use different MPA thresholds, different limitations on food group intakes. | PA and MPA using usual intake, as described by Joseph & Carriquiry^(16)^. | 11 micronutrients: Vitamin A, vitamin C, thiamine, riboflavin, niacin, vitamin B6, folate, vitamin B12, calcium, iron and zinc. | Different food group performance indicators were investigated using 6, 9, 13 and 21 food groups. The indicators also vary in regard to the amount of food eaten (either 1 gram (g) or 15 g) that must be consumed in order for the food group to count. | Performance of the indicators was assessed using ROC analysis, and through examination of indicator characteristics such as sensitivity, specificity and total misclassification.  In this case, sensitivity assesses the proportion of all those who truly have better MPA who are identified by the indicator. | Across all three indicators and all three MPA thresholds, the same food group cutoff of ≥ five provides the best balance between sensitivity and specificity while minimizing misclassification. The sensitivity at MPA threshold of 50% may be unacceptably low for food groups 6 and 9. At higher MPA thresholds (60% and 70 %), The 9 food group indicator provides substantially better sensitivity than 6. and higher specificity than 13. |

DDS: dietary diversity score; NAR: Nutrition adequacy ratio; MAR: mean adequacy ratio**;** RDA: recommended dietary allowance; ROC: Receiver Operating Characteristics; NFCS: National Food Consumption Survey; FAO: Food and Agriculture Organisation; DRIs: Dietary Recommended Intakes; CV: Coefficient of variation; IOM: Institute of Medicine; SDs: Standard Deviations; SLR Model: Simple linear regression; WDDP: Women’s Dietary Diversity Project; BLUP: Best linear unbiased predictor; NCR Method: National Research Council Method.

**Supplementary S.2**

EAR for Zinc

The EARs for zinc were calculated assuming high bioavailability^(32)^. These figures are based on FAO/WHO 2002 recommended nutrient intakes^(32)^ and adapted from Allen *et al.*^(33)^, although given as 2.2, 4.0 and 7.0 by the IOM^(34)^.

Table S.2: EARs for Zinc and calculated EARs for zinc with high bioavailability for selected life-stage groups

| Age (years) | IOM^(34)^ | Gibson and Ferguson*^(32)^ | Used in this study |
| --- | --- | --- | --- |
| 1-3 years | 2.2 | 2.0 | 2.0 |
| 4-6 years | 4.0 | 2.4 | 2.4 |
| 7-8 years | 4.0 |  | 2.4 |
| 9 years | 7.0 |  | 2.4 |

*High bioavailability

**Supplementary S.3**: Supplementary S.3 provides the EAR/AI values for the 15 micronutrients used to calculate nutrient adequacy for Method 1, as well as the additional CV values to calculate the PA-P and PA-U for Methods 2 and 3. The PROBNORM function is SAS is as follows:

PA=PROBNORM(estimated child’s intake - EAR)/(CV.EAR).

The CV values used were 15% for niacin and 25% for zinc, 20% for vitamin A and 10% for the rest^(14,34)^. The assumption is that the nutrients have a normal distribution except for calcium and iron. Using the assumed normal distributions, the “PROBNORM” function in SAS (SAS Institute Inc., Cary, NC, USA.) can be used to calculate the probability of adequacy.

Table S.3: EARs used for the fixed cut-point method (Method 1) and used to calculate the probabilities of adequacy for Method 2, with additional CV values for Method 2

| Nutrient | EAR (1-3 years) | EAR (4-8 years) | EAR (9 years) | CV^†^ | Lambda |
| --- | --- | --- | --- | --- | --- |
| Calcium (AI) | 500.0 | 800.0 | 1300.0 | Supple-mentary S.4 | 0.20 |
| Iron | 3.0 | 4.1 | 5.9 (M) /  5.7 (F) | Supple-mentary S.5 | 0.25 |
| Magnesium | 65.0 | 110.0 | 200.0 | 10% | 0.30 |
| Phosphorus | 380.0 | 405.0 | 1055.0 | 10% | 0.35 |
| Zinc* | 2.0 | 2.4 | 2.4 | 25%^††^ | 0.25 |
| Vitamin A | 210.0 | 275.0 | 445.0 (M) / 420.0 (F) | 20% | -0.10 |
| Vitamin C | 13.0 | 22.0 | 39.0 | 10% | 0.30 |
| Vitamin E | 5.0 | 6.0 | 9.0 | 10% | 0.15 |
| Folate | 120 | 160 | 250 | 10% | 0.10 |
| Niacin | 5.0 | 6.0 | 9.0 | 15% | 0.35 |
| Pantothenic acid (AI) | 2.0 | 3.0 | 4.0 | 10% | 0.20 |
| Riboflavin | 0.4 | 0.5 | 0.8 | 10% | 0.15 |
| Thiamine | 0.4 | 0.5 | 0.7 | 10% | 0.25 |
| Vitamin B6 | 0.4 | 0.5 | 0.8 | 10% | 0.30 |
| VitaminB12 | 0.7 | 1.0 | 1.5 | 10% | 0.20 |

EAR/AI:^(34)^.

*Zinc: EARs high bioavailability (Reference-Table 10.2, Gibson and Ferguson^(32)^) – see **Supplementary S.2.**.

^†^CV: IOM 2006 ^(34)^.

^††^CV Zinc 25%, FAO/WHO. Human and vitamin mineral requirements. Rome: FAO/WHO^(35)^.

2002. Report of a Joint FAO/WHO expert consultation, referred to in Kennedy *et al.*^(14)^.

**Supplementary S.4**

Probability of adequacy for calcium

Foote *et al.*^(13)^ remarked that the AI for calcium is available, but that no SDs have been specified. The probability of adequacy of calcium intake is calculated by comparing intake levels to the AI. The PA for calcium is calculated as follows:

PA=0% for calcium intake ≤ 25% of AI;

PA=25% for 25% of AI < calcium intake ≤ 50% of AI;

PA=50% for 50% of AI < calcium intake ≤ 75% of AI;

PA=75% for 75% of AI < calcium intake ≤ 100% of AI; and

PA=100% for calcium intake > AI.

**Supplementary S.5**

EAR for iron

The PA, or 1-(probability of inadequacy) for iron was calculated assuming high bioavailability^(32)^. For the purpose of assessing populations, a probability of 1 has been assigned to usual intakes that are below the 2.5th percentile of requirements, and a probability of 0 has been assigned to usual intakes that fall above the 97.5th percentile of requirements. Foote *et al.*^(13)^ noticed that the distribution of iron is skew, and proposed the use of published tables to establish probabilities of inadequacy.

Table S.5: Probability of inadequate iron intakes for different age groups at different ranges of usual intake^(32)^

| Probability of inadequacy* | Usual intake of children 1-3 years | Usual intake of children 4-8 years |
| --- | --- | --- |
| 1.0 | <1.3 | <1.6 |
| 0.96 | 1.3 – 1.5 | 1.6 – 2.0 |
| 0.93 | 1.5 – 1.8 | 2.0 – 2.4 |
| 0.85 | 1.8 – 2.4 | 2.4 – 3.2 |
| 0.75 | 2.4 – 2.8 | 3.2 – 3.8 |
| 0.65 | 2.8 – 3.2 | 3.8 – 4.3 |
| 0.55 | 3.2 – 3.6 | 4.3 – 4.9 |
| 0.45 | 3.6 – 4.1 | 4.9 – 5.6 |
| 0.35 | 4.1 – 4.6 | 5.6 – 6.3 |
| 0.25 | 4.6 – 5.3 | 6.3 – 7.3 |
| 0.15 | 5.3 – 6.3 | 7.3 – 8.8 |
| 0.08 | 6.3 – 7.3 | 8.8 – 10.1^†^ |
| 0.04 | 7.3 – 8.2 | 10.1 – 11.7^†^ |
| 0.0 | >8.2 | >11.7^†^ |

*High bioavailability

^†^Values adapted from Table 10.4 in Gibson and Ferguson^(32)^.

**Supplementary S.6**

Steps taken to calculate the probability of adequacy using usual intakes (PA-U) and the mean probability of adequacy using usual intakes (MPA-U) (Method 3):

Method 3 is described by Arimond *et al.*^(30)^, usual intake, Day-1 intake plus two additional intakes for a representative sample.

Step 1: Use PROC TRANSREG in SAS to estimate, using a Box-Cox transformation, for each nutrient a λ-value to provide the closest fit to a normal distribution. These λ-values are listed in **Table A.1**. Transform the nutrient intakes ($x_{i})$using the Box-Cox power transformation to obtain $y_{i}$with the appropriate parameter (λ) to approximate normal, using the following formula: $y_{i}=({x_{i}}^{\lambda}-1)/\lambda$.

Step 2:

2.1 Calculate individual and population means for intakes of each nutrient using the transformed variables $Y_{i}$ (note that some individuals had only one observation), obtaining $\bar{Y}_{i.}$ and $\bar{Y}_{..}$ and $n_{i}$, the number of surveys for subject $i$.

2.2 Calculate within- and between-individual variances for the transformed intake variables, obtaining $\sigma_{ww}$ and $\sigma_{yy}$.

2.3 Using these variances, the “best linear unbiased predictor” (BLUP) of the usual intake for each nutrient for each subject $i$ is calculated as

$$\bar{y}_{i}=\bar{Y}_{..}+\sqrt{\frac{\sigma_{yy}}{\left( \sigma_{yy}+\frac{\sigma_{ww}}{n_{i}} \right)}}(\bar{Y}_{i.}-\bar{Y}_{..})$$

Compute these BLUP-values for each subject and for each of the 13 nutrients, excluding calcium and iron^(15)^.

Step 3:

Using the BLUPs, calculate the PA for iron from the Table S.5. Also calculate the PA for calcium using the method of Foote *et al.*^(13)^ (also described in Supplementary S.4).

Step 4:

With the exception of calcium and of iron, information on the distribution of requirements (CVs and EARs (or AIs)) is available and distributions are assumed to be approximately normal. For these remaining 13 nutrients transform the requirement distributions using the same power transformation as selected above for each nutrient. This is done by generating random normal variables to simulate the requirement distribution; this distribution was then Box-Cox transformed.

Therefore, for each nutrient:

Step 4.1: Generate 800 normal random variables, associated with each subject, use the age and gender related EAR (as mean value) and SD, (SD=CV*EAR/100). See Table S.3^(30)^.

Step 4.2: For each nutrient, use the λ-value obtained in Step 1 and the simulated normal random variables (${nut\_gen}_{ij})$to generate for each subject, 800 simulated Box-Cox-transformed values ${nut\_gen\_bc}_{ij}$ for subject $i$ and simulation number $j,$using the formula ${nut\_gen\_bc}_{ij}={(nut\_gen}_{ij}^{\lambda}-1)/\lambda$.

Step 4.3: Use the 800 simulated values per subject, determine the number of generated Box-Cox values smaller than the BLUP-values generated in Step 2.

Repeat this process for each of the 13 nutrients.

**Step 5**: The PA per subject for each nutrient is then calculated as the proportion (out of 800) obtained in step 4.3. Average the 15 PA values, including iron and calcium, to obtain MPA. The distribution of MPA is also transformed to approximate normality. Untransformed values are presented in descriptive tables, and the transformed variable was used in correlation and regression analyses.

**Supplementary S.7**

Table S7.1: Evaluation of minimum dietary diversity (MDD) indicators for different MAR (Method 1) thresholds for data from the example dataset for 2 - <6-year-old children

|  | | MAR thresholds^‡^ for Method 1: age 2 - <6 years (n=691) | | |
| --- | --- | --- | --- | --- |
|  | | ≥0.6^¶^ | ≥0.7 | ≥0.8 |
| n (%) (≥threshold or prevalence of adequate intake) | | 679  (98.3%) | 663  (95.9%) | 594  (86.0%) |
| Logistic regression:  (Odds ratio (95% CI) | | 4.55***  (2.23-9.31) | 4.21***  (2.57-6.89) | 3.52***  (2.64-4.70) |
| AUC (95% CI) | | 0.824^†††^  (0.709-0.938) | 0.805^†††^  (0.727-0.883) | 0.779^†††^  (0.732-0.826) |
| MDD=3^§^ | Sensitivity | 0.837 | 0.846 | 0.884 |
|  | Specificity | 0.667 | 0.607 | 0.515 |
|  | Youden index | **0.503** | **0.453** | **0.399** |
|  | Euclidean distance | **0.371** | **0.422** | 0.499 |
|  | LR+ (post probability of TH) | 2.51 (0.79) | 2.15 (0.83) | 1.82 (0.88) |
|  | Accuracy | **0.834** | **0.836** | **0.832** |
|  | Kappa | **0.063** | **0.165** | **0.374** |
| MDD=4 | Sensitivity | 0.498 | 0.507 | 0.545 |
|  | Specificity | 0.917 | 0.893 | 0.845 |
|  | Youden index | 0.415 | 0.400 | 0.390 |
|  | Euclidean distance | 0.509 | 0.504 | **0.481** |
|  | LR+ (post probability of TH) | 5.97 (0.90) | 4.73 (0.92) | 3.53 (0.93) |
|  | Accuracy | 0.505 | 0.522 | 0.588 |
|  | Kappa | 0.027 | 0.068 | 0.235 |

LR+: Positive Likelihood Ratio Test; TH: Threshold;. ROC: Receiver operating characteristic; AUC: Area under the ROC curve; MDD: Minimum dietary diversity; MAR: Mean adequacy ratio.

Significant odds ratio, ***p<0.001; Test that AUC=0.5, Wald Chi-square test for ROC contrast, ^†††^p<0.001.

^‡^Threshold levels <0.6 were excluded, n=2.

^§^Select the MDD indicator for each threshold using the maximum Youden index.

^¶^The best threshold is ≥0.6, using the maximum AUC over all thresholds.

Table S7.2: Evaluation of MDD indicators for different MAR (Method 1) thresholds for data from the example dataset for 6 - <10-year-old children

|  | | MAR thresholds^‡^ for Method 1: age 6-<10 years (n=479) | | |
| --- | --- | --- | --- | --- |
|  | | ≥0.6^¶^ | ≥0.7 | ≥0.8 |
| n (%) (≥threshold or prevalence of adequate intake) | | 471  (98.3%) | 455  (95.0%) | 410  (85.6%) |
| Logistic regression:  (Odds ratio (95% CI) | | 4.99**  (1.79-13.89) | 3.42***  (1.97-5.93) | 2.58***  (1.88-3.52) |
| AUC (95% CI) | | 0.798^††^  (0.636-0.960) | 0.769**^†††^**  (0.684-0.855) | 0.731**^†††^**  (0.671-0.791) |
| MDD=3^§^ | Sensitivity | 0.826 | 0.837 | 0.863 |
|  | Specificity | 0.625 | 0.542 | 0.449 |
|  | Youden index | **0.451** | **0.379** | 0.312 |
|  | Euclidean distance | **0.413** | **0.486** | 0.568 |
|  | LR+ and post probability of TH | 2.20 (0.77) | 1.83 (0.81) | 1.57 (0.86) |
|  | Accuracy | **0.823** | **0.823** | **0.804** |
|  | Kappa | **0.075** | **0.181** | **0.271** |
| MDD=4 | Sensitivity | 0.482 | 0.495 | 0.524^§^ |
|  | Specificity | 0.875 | 0.875 | 0.812^§^ |
|  | Youden index | 0.357 | 0.370 | **0.336** |
|  | Euclidean distance | 0.533 | 0.520 | **0.512** |
|  | LR+ and post probability of TH | 3.86 (0.85) | 3.96 (0.90) | 2.78 (0.92) |
|  | Accuracy | 0.489 | 0.514 | 0.566 |
|  | Kappa | 0.025 | 0.069 | 0.178 |

LR+: Positive Likelihood Ratio Test; TH: Threshold;. ROC: Receiver operating characteristic; AUC: Area under the ROC curve; MDD: Minimum dietary diversity; MAR: Mean adequacy ratio.

Significant odds ratio, **p<0.01, ***p<0.001; Wald Chi-square test for ROC contrast, ^††^p<0.001, ^†††^p<0.001.

^‡^Threshold levels <0.6 were excluded, n=8 (an exception on the limit of 10).

^§^Select the MDD indicator for each threshold using the maximum Youden index.

^¶^The best threshold is 0.6, using the maximum AUC over all thresholds.

Table S7.3: Evaluation of MDD indicators for different MPA-P (Method 2) thresholds for data from the example dataset for 2 - <6-year-old children

|  | | MPA-P thresholds^‡^ for Method 2: age 2-<6 years (n=691) | | | |
| --- | --- | --- | --- | --- | --- |
|  | | ≥0.5^¶^ | ≥0.6 | ≥0.7 | ≥0.8 |
| n (%) (≥threshold or prevalence of adequate intake) | | 624  (90.3%) | 577  (83.5%) | 495  (71.6%) | 359  (52.0%) |
| Logistic regression:  Odds ratio (95% CI) | | 2.55***  (1.90-3.41) | 2.09***  (1.67-2.61) | 2.12***  (1.76-2.55) | 2.01***  (1.71-2.37) |
| AUC (95% CI) | | 0.727^†††^  (0.668-0.786) | 0.684^†††^  (0.634-0.734) | 0.688^†††^  (0.646-0.729) | 0.682^†††^  (0.644-0.719) |
| MDD=3 | Sensitivity | 0.857 | 0.865 | 0.893 | 0.928 |
|  | Specificity | 0.448 | 0.360 | 0.337 | 0.280 |
|  | Youden index | 0.305 | 0.225 | 0.230 | 0.208 |
|  | Euclidean distance | 0.570 | 0.654 | 0.672 | 0.724 |
|  | LR+ and post probability of TH | 1.55 (0.61) | 1.35 (0.67) | 1.35 (0.76) | 1.29 (0.84) |
|  | Accuracy | **0.818** | **0.782** | **0.735** | 0.616 |
|  | Kappa | **0.255** | **0.247** | **0.297** | 0.228 |
| MDD=4^§^ | Sensitivity | 0.521 | 0.532 | 0.562 | 0.610 |
|  | Specificity | 0.791 | 0.719 | 0.689 | 0.639 |
|  | Youden index | **0.312** | **0.251** | **0.250** | **0.249** |
|  | Euclidean distance | **0.523** | **0.546** | **0.536** | **0.531** |
|  | LR+ and post probability of TH | **2.49 (0.71)** | **1.90 (0.74)** | **1.80 (0.81)** | **1.69 (0.87)** |
|  | Accuracy | 0.547 | 0.563 | 0.598 | **0.624** |
|  | Kappa | 0.120 | 0.182 | 0.264 | **0.267** |

LR+: Positive Likelihood Ratio Test; TH: Threshold;. ROC: Receiver operating characteristic; AUC: Area under the ROC curve; MDD: Minimum dietary diversity; MPA-P: Mean probability of adequacy using the probability method.

Significant odds ratio, **p<0.01, ***p<0.001; Wald Chi-square test for ROC contrast, ^††^p<0.001, ^†††^p<0.001.

^‡^Threshold levels <0.5 were excluded..

^§^Select the MDD indicator for each threshold using the maximum Youden index.

^¶^The best threshold is 0.5, using the maximum AUC over all thresholds.

Table S7.4: Evaluation of MDD indicators for different MPA-P (Method 2) thresholds for data from the example dataset for 6 - <10-year-old children

|  | | MPA-P thresholds^‡^ for Method 2: age 6-<10 years (n=479) | | | |
| --- | --- | --- | --- | --- | --- |
|  | | ≥0.5^¶^ | ≥0.6 | ≥0.7 | ≥0.8 |
| n (%) (≥threshold or prevalence of adequate intake) | | 430  (89.8%) | 387  (80.8%) | 323  (67.4%) | 213  (44.5%) |
| Logistic regression:  Odds ratio (95% CI) | | 2.88***  (1.97-4.19) | 2.00***  (1.55-2.57) | 2.00***  (1.62-2.47) | 2.31***  (1.88-2.83) |
| AUC (95% CI) | | 0.746^†††^  (0.683-0.809) | 0.676^†††^  (0.618-0.734) | 0.681^†††^  (0.634-0.728) | 0.725^†††^  (0.681-0.768) |
| MDD=3 | Sensitivity | 0.851 | 0.866 | 0.882 | 0.930 |
|  | Specificity | 0.469 | 0.380 | 0.314 | 0.271 |
|  | Youden index | 0.320 | **0.246** | 0.196 | 0.201 |
|  | Euclidean distance | 0.552 | 0.634 | 0.696 | 0.732 |
|  | LR+ and post probability of TH | 1.60 (0.62) | 1.40 (0.68) | 1.29 (0.75) | 1.27 (0.84) |
|  | Accuracy | **0.812** | **0.773** | **0.697** | 0.564 |
|  | Kappa | **0.197** | **0.221** | 0.235 | 0.221 |
| MDD=4^§^ | Sensitivity | 0.512 | 0.517 | 0.560 | 0.676 |
|  | Specificity | 0.837 | 0.696 | 0.699 | 0.684 |
|  | Youden index | **0.348** | 0.213 | **0.259** | **0.360** |
|  | Euclidean distance | **0.515** | **0.571** | **0.533** | **0.452** |
|  | LR+ and post probability of TH | 3.13 (0.76) | 1.70 (0.72) | 1.86 (0.81) | 2.14 (0.90) |
|  | Accuracy | 0.545 | 0.551 | 0.606 | **0.681** |
|  | Kappa | 0.118 | 0.108 | **0.272** | **0.393** |

LR+: Positive Likelihood Ratio Test; TH: Threshold;. ROC: Receiver operating characteristic; AUC: Area under the ROC curve; MDD: Minimum dietary diversity; MPA-P: Mean probability of adequacy using the probability method.

Significant odds ratio, **p<0.01, ***p<0.001; Wald Chi-square test for ROC contrast, ^†††^p<0.001.

^‡^Threshold levels <0.5 were excluded.

^§^Select the MDD indicator for each threshold using the maximum Youden index.

^¶^The best threshold is 0.5, using the maximum AUC.

**Supplementary S.8**

Demonstration of the calculation of sensitivity and specificity for identification of a possible MDD indicator the left or the right of the median of the dietary diversity scores, using Method 1 and Method 2:


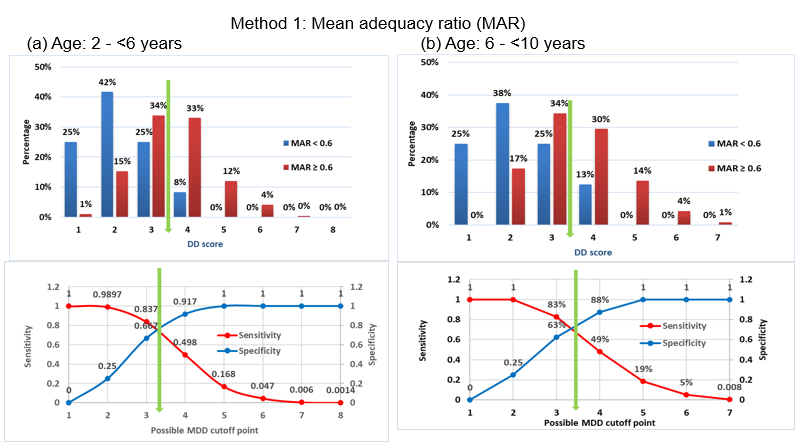


| **MDD≥3:**  Sensitivity=34+33+12+4+0 =83%  Specificity=25+42=67%  **MDD≥4:**  Sensitivity=33+12+4+0=49%  Specificity=25+42+25=92% | **MDD≥3:**  Sensitivity=34+30+14+4+1 =83%  Specificity=25+38=63%  **MDD≥4:**  Sensitivity=30+14+4+1=49%  Specificity=25+38+25=88% |
| --- | --- |
|  |  |

Figure S.8.1 (a),(b): Demonstration of the calculation of sensitivity and specificity for identification of a possible minimum dietary diversity (MDD) indicator to the left and right of the median of the dietary diversity (DD) scores, using a mean adequacy ratio (MAR) threshold of 0.6 (Method 1)

MAR: Mean adequacy ratio; DD: Dietary diversity; MDD: Minimum dietary diversity.


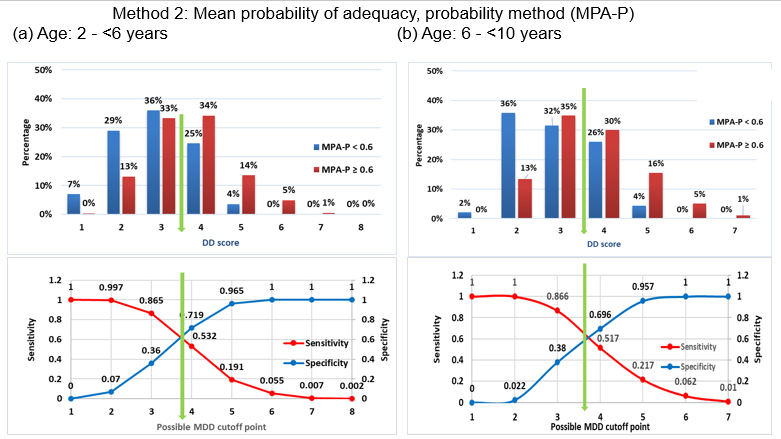


| **MDD≥3:**  Sensitivity=33+34+14+5+1 =87%  Specificity=7+29=36%  **MDD≥4:**  Sensitivity=34+14+5+1=54%  Specificity=7+29+36=72% | **MDD≥3:**  Sensitivity=35+30+16+5+1 =87%  Specificity=2+36=38%  **MDD≥4:**  Sensitivity=30+16+5+1=52%  Specificity=2+36+32=70% |
| --- | --- |

Figure S.8.2 (a),(b): Demonstration of the calculation of sensitivity and specificity for minimum dietary diversity (MDD) indicators to the left and right of the median of the dietary diversity (DD) scores using a mean probability of adequacy using the probability method (MPA-P) threshold of 0.6 (Method 2)

MPA-P: Mean probability of adequacy using the probability method; DD: Dietary diversity; MDD: Minimum dietary diversity.

**Supplementary S.9**

Investigation of MDD indicators in other studies:

Table S.9.1: The comparison of the selected maximum dietary diversity (MaxDD) indicator with the mean DD scores for different studies, with the focus on inadequate nutrient intake

| Selected studies investigating inadequacy: (in these cases sensitivity > specificity, sensitivity identifies inadequacy) | | | | | | |
| --- | --- | --- | --- | --- | --- | --- |
| Study | MAR/ MPA-P/ MPA-U | Mean DD scores  (number of food groups) | Mean adequacy | Inadequacy threshold selected | MaxDD indicator selected (sensitivity, specificity) | Cutoff point selection preference |
| Schuette  *et al.*^(24)^  (n=2489 College students) | MAR  (RDA) | 4.04 (5) | 0.82 | 0.75 | ≤5  (89%, 45%) | Preference to high sensitivity |
| Hatløy *et al.*^(5)^  (n=77 Mali children, 13-58 months) | MAR  (RDA) | 5.8 (8) | 0.77 | 0.75 | ≤6  (77%, 33%) | Preference to sensitivity>75%, specificity>30% |
| Steyn *et al.*^(6)^  (n=2200 South African children,1-9 years) | MAR  (RDA) | 3.6 (9) | 0.63 | 0.70 | ≤4  (75%, 70%) | Balance between sensitivity and specificity |
| Steyn *et al.*^(36)^  (n=2200 South African children, 1-9 years) | MAR  (EAR) | 3.8 (9) | 0.63 | 0.5 | ≤4  (88%, 57%) | Higher sensitivity, lowest misclassification |
| Kennedy  *et al.*^(14)^  (n=2805 Fillipino children, 24-71 months) | MPA-P  (EAR) | 4.91 (9) | 0.33 | 0.5 | ≤5  (curves meet) | Balance between sensitivity and specificity |
| Zhao *et al.*^(28)^  (n=1694 Chinese children, 3-12 years) | MPA-P  (EAR) | 6.3 (9) | 0.39 | 0.75 | Between 7 and 8  (curves meet) | Balance between sensitivity and specificity |
| Torrico^(37)^  (n=7448 Filipino children, 3-18 years) | MAR  (EAR) | 6.18  (10) | 0.69 | 0.5 & 0.75 | ≤6 (74%, 45%)  & ≤7 (55%, 68%) | Highest sensitivity and specificity |

DD: Dietary diversity; MaxDD: Maximum dietary diversity indicator; MAR: Mean adequacy ratio; MPA-P: Mean probability of adequacy using the probability method; MPA-U: Mean probability of adequacy using usual intakes; RDA: Recommended dietary allowance; EAR: Estimated average requirement

Table S.9.2: The comparison of the selected minimum dietary diversity (MDD) indicator with the mean DD scores for different studies, with the focus on adequate nutrient intake

| Selected studies investigating adequacy: (sensitivity identifies adequate intake, in these cases sensitivity > specificity) | | | | | | |
| --- | --- | --- | --- | --- | --- | --- |
| Study | MPA-P/ MPA-U | Mean DD score  (number of food groups) | Mean adequacy | Adequacy threshold selected | MDD indicator selected (sensitivity, specificity) | Cutoff point selection preference |
| Rani^(29)^  (n=232 children, 5-8 years, rural India | MPA-P | 6.5 (13 sub-food groups) | 0.40 | 0.5 | Between 6 and 7 (sensitivity >60%, specificity>30%) | Prefer sensitivity |
| Arimond *et al.*^(30)^  (n=130 NPNL women, Burkina Faso) | MPA-U | 4.3  (FGI-9R) | 0.39 | 0.5 | ≥5  (67%, 66%)  (same as Youden) | Prefer both sensitivity and specificity above 60%, see footnote^†^ |
| Arimond *et al.*^(30)^  (n=102 NPNL women, Mali) | MPA-U | 4.4  (FGI-9R) | 0.47 | 0.5 | ≥5^†^  (62%, 75%)  (same as Youden) |  |
| Arimond *et al.*^(30)^  (n=103 NPNL women, Mozambique) | MPA-U | 3.7  (FGI-9R) | 0.54 | 0.5 | ≥4^†^  (70%, 60%)  (same as Youden) |  |
| Arimond *et al.*^(30)^  (n=299 NPNL women, Bangladesh) | MPA-U | 3.6  (FGI-9R) | 0.35 | 0.5 | ≥5^†^*  (44%, 85%)  (Youden ≥4) |  |
| Arimond *et al.*^(30)^  (n=1798 NPNL women, Philippines ) | MPA-U | 3.3  (FGI-9R) | 0.34 | 0.5 | ≥4^†^  (57%, 65%)  (same as Youden) |  |
| Caswell *et al.*^(31)^  (n=200 rural children, Zambia 4-8 years) | MPA-U | 4.1  (7 DDS-IYC) | 0.75 | 0.75 | ≥4  (99%, 5%) for late post-harvest season,  (79%, 32%) for early lean season , (71%, 76%) for  late lean season | Sensitivity and specificity reported for WHO recommended cutoffs. |
|  |  | 4.4  (10 DDS-W) | 0.75 | 0.75 | ≥5  (86%, 30%) for  the late post-harvest season,(58%,69%) for early lean season,  and (71% and 65%) for late lean season |  |
| Diop *et al.*^(19)^  (n=1066 Burkina Faso children, 24-59 months) | MPA-U | 3.1  (7 DDS-IYC) | 0.58 | 0.75 | ≥4  (56%,79%) | Good specificity and moderate sensitivity |
|  |  | 3.4  (10 DDS-W) | 0.58 | 0.75 | ≥4 (76%, 70%) | Optimized sensitivity and specificity |
| Monge-Rojas *et al.*^(38)^ (n=818 and 1202 Costa Rico and Mexico adolescents, 1318 years) | MPA-U | 4.2 and 4.7  (10 DDS-W) | 0.77; 0.76 | 0.70 | ≥4,  ( 71%, 64%) and for ≥5  (63%, 65%) | Youden index |

DD: Dietary diversity; MDD: Minimum dietary diversity; WHO: World Health Organization; MPA-P: Mean probability of adequacy using the probability method; MPA-U: Mean probability of adequacy using usual intakes.

*Bangladesh represented one extreme, with 86 percent of energy intakes accounted for by starchy staples (almost exclusively polished rice). Even if there may have been some overestimation of rice and total energy intakes, reported quantities of all other foods were very small and it is likely that diets at this site would still be the most monotonous among the five sites examined^(30)^. If the Youden index is used the MDD indicator is 4.

^†^To select the MDD indicator, they considered criteria such as sensitivity, specificity (preferably both ≥ 60 percent; still considered if one of the two only is ≥ 50 percent) and the rate of misclassification (preferably ≤30 percent; still considered if ≤ 40 percent).

NPNL: Non-pregnant, non-lactating.

FGI-9R: Food group diversity indicator comprised of 9 food groups that impose a 15g minimum intake.

DDS-IYC: 7-food group score for assessing infant and young child feeding.

DDS-W: 10-food group score for use among women with reproductive age.

Table S.9.3: Recalculation of the indicator characteristics for the food group diversity indicator using 9 restricted food groups with the threshold of the mean probability of adequacy using usual intakes (MPA-U)>0.5, from Appendix 3 in Arimond *et al.*^(30)^

| Country | Total n (L_50_) | Recalculated MDD indicator | Mean (SD) | Count | Sensitivity and specificity^(30)^ | | Maximum Youden index |
| --- | --- | --- | --- | --- | --- | --- | --- |
| Burkina Faso | 130  (65.5) | **≥5**  <5 | 4.3  (1.1) | 56  74 | 66.7 | 66.0 | 0.327 |
| Mal | 102  (51.5) | **≥5**  <5 | 4.4  (1.1) | 43  59 | 61.7 | 74.5 | 0.362 |
| Mozambique | 103  (52.0) | **≥4**  <4 | 3.7  (0.8) | 60  43 | 69.8 | 60.0 | 0.298 |
| Bangladesh | 299  (150.0) | **≥4**  <4 | 3.6  (1.1) | 160  139 | 83.1 | 53.8 | 0.369 |
| Philippines | 1798  (899.5) | **≥4**  <4 | 3.3  (1.1) | 729  1069 | 56.8 | 65.4 | 0.222 |

L_50_: Position of the median, $L_{50}=\frac{\left( n+1 \right)50}{100}$.

MDD: Minimum dietary diversity; SD: Standard deviation.

**References**

1. Madden JP & Yoder MD (1972) Program evaluation: Food stamps and commodity

distribution in rural areas of central Pennsylvania. *Pennsylvania Agricultural*

*Experiment Station Bulletin* **78**, 1-119.

1. Guthrie HA & Scheer JC (1981) Validity of a dietary score for assessing

nutrient adequacy. *J. Am. Diet. Assoc.* **78,** 240-245.

1. Madden JP, Goodman SJ, Guthrie HA (1976) Validity of the 24-hr recall. Analysis of data obtained from elderly subjects. *J. Am. Diet Assoc.* **68**, 143-147.
2. Krebs-Smith SM, Smiciklas-Wright H, Guthrie HA *et al.* (1987) The effects of variety in food choices on dietary quality. *J. Am. Diet. Assoc.* **87,** 897-902.
3. Hatløy A, Torheim LE, Oshaug A (1998) Food variety - a good indicator of nutritional adequacy of the diet? A case study from an urban area in Mali, West Africa. *Eur J Clin Nutr* **52**(12), 891-8. doi: 10.10ries38/sj.ejcn.1600662. PMID: 9881884.
4. Steyn N, Nel J, Nantel G, *et al.* (2006) Food variety and dietary diversity scores: are they good indicators of dietary adequacy? *Public Health Nutr*. **9**, 644–50.
5. NRC (National Research Council) (1986) *Nutrient adequacy: Assessment using food consumption surveys*. Food and Nutrition Board, National Academy Press, Washington, D.C.
6. Institute of Medicine (2003) *Dietary reference intakes*. Food and Nutrition Board. Washington, DC: National Academies Press.
7. Oldewage-Theron W, Kruger R (2011) Dietary diversity and adequacy of women caregivers in a peri-urban informal settlement in South Africa. *Nutrition* **27**, 420–427.
8. Islam MH, Jubayer A, Nowar A *et al.* (2023) Dietary diversity and micronutrients adequacy among the women of reproductive age at St. Martin’s island in Bangladesh. *BMC Nutrition* ***9****, 52.* Published online: 21 March 2023. https://doi.org/10.1186/s40795-023-00715-y.
9. Zhong W, Zhao A, Lan H *et al.* (2022) Dietary Diversity, Micronutrient Adequacy and Bone Status during Pregnancy: A Study in Urban China from 2019 to 2020. *Nutrients* **14**, 4690. Published online: 5 November 2022. https://doi.org/10.3390/nu14214690.
10. Arimond M & Ruel MT (2004) *Dietary Diversity, Dietary Quality, and Child Nutritional Status: Evidence from Eleven Demographic and Health Surveys*. Washington, D.C.: Food and Nutrition Technical Assistance (FANTA) Project, Academy for Educational Development (AED).
11. Foote JA, Murphy SP, Wilkens LR, Basiotis PP *et al.* (2004) Dietary variety increases the probability of nutrient adequacy among adults. *American Society for Nutritional Sciences, J Nutr.* **134**, 1779-1785.
12. Kennedy G, Pedro MR, Seghieri C *et al.* (2007) Dietary diversity score is a useful indicator of micronutrient intake in non breast-feeding Filipino children. *Journal of Nutrition* **137**, 1-6.
13. Institute of medicine (IOM) (2000) *Dietary Reference Intakes: Applications in Dietary Planning / Subcommittee on Interpretation and Uses of Dietary Reference Intakes and the Standing Committee on the Scientific Evaluation of Dietary Reference Intakes.* Washington DC: National Academic Press.
14. Joseph M & Carriquiry A (2010) A measurement error approach to assess the association between dietary diversity, nutrient intake, and mean probability of adequacy. *J Nutr*. **140**(11), 2094S - 101S.
15. Martin-Prevel Y, Allemand P, Wiesmann D *et al*. (2015) Moving Forward on Choosing a Standard Operational Indicator of Women’s Dietary Diversity (Internet). Rome: FAO; 2015. Available from: <http://www.fao.org/3/i4942e/i4942e.pdf>. (accessed July 2024).
16. Arsenault JE, Yakes EA, Islam MM *et al.* (2013) Very low adequacy of micronutrient intakes by young children and women in rural Bangladesh is primarily explained by low food intake and limited diversity. J Nutr **143**,197–203.
17. Diop L, Becquey E, Turowska Z *et al.* (2021) Standard Minimum Dietary Diversity Indicators for Women or Infants and Young Children Are Good Predictors of Adequate Micronutrient Intakes in 24-59-Month-Old Children and Their Nonpregnant Nonbreastfeeding Mothers in Rural Burkina Faso. J. Nutr. **151**, 412–422. (CrossRef) (PubMed).
18. Nusser SM, Carriquiry AL, Dodd KW *et al.* (1996) A semi-parametric transformation approach to estimating usual nutrient intake distributions. *J. Am. Stat. Assoc.* **91**, 1440–1449.
19. Tooze JA, Midthune D, Dodd KW, *et al.* (2006) A new statistical method for estimating the usual intake of episodically consumed foods with application to their distribution. *J Am Diet Assoc* **106**, 1575–1587.
20. Page L & Phippard E (1957) *Essentials of an adequate diet, facts for nutrition programs*. USDA, Home Economics Research Report No. 3, 1957.
21. Better Eating for Better Health (1984) Washington, DC: American Red Cross, 1984.
22. Schuette LK, Song WO & Hoerr SL (1996) Quantitative use of the Food Guide Pyramid to evaluate dietary intake of college students. *J Am Diet. Assoc.* **96**, 453-457.
23. Food Guide Pyramid: A Guide to Daily Food Choices (1992) Washington, DC: US Dept of Agriculture, Human Nutrition Information Service. Home and Garden Bulletin No 252.
24. Ruel M, Graham J, Murphy S *et al.* (2004) *Validating simple indicators of dietary diversity and animal source food intake that accurately reflect nutrient adequacy in developing countries*. Report submitted to GL-CRSP, 2004.
25. Oldewage-Theron WH & Kruger R (2008) Food Variety and Dietary Diversity as Indicators of the Dietary Adequacy and Health Status of an Elderly Population in Sharpeville, South Africa. *Journal of Nutrition for the Elderly* **27**:1-2, 101-133, doi: 0.1080/01639360802060140 To link to this article: <https://doi.org/10.1080/01639360802060140>.
26. Zhao W, Yu K, Tan S *et al*. (2017) Dietary diversity scores: an indicator of micronutrient inadequacy instead of obesity for Chinese children. *BMC Public Health*. **17**,440. doi: 10.1186/s12889-017-4381-x.
27. Rani V (2010) Dietary diversity as an indicator of micronutrient adequacy of the diet of five to eight year old Indian rural children. *Nutrition & Food Science* **40**(5), 466-476. DOI 10.1108/00346651011076974.
28. Arimond M, Wiesmann D, Becquey E *et al*. (2011) *Dietary Diversity as a Measure of the Micronutrient Adequacy of Women’s Diets in Resource-Poor Areas: Summary of Results from Five Sites*. Washington, DC: FANTA-2 Bridge, FHI 360.
29. Caswell BL, Talegawkar SA, Siamusantu W *et al.* (2018) A 10-food group dietary diversity score outperforms a 7-food group score in characterizing seasonal variability and micronutrient adequacy in rural Zambian children. *J Nutr*. **148**(1), 131–9.
30. Gibson RS & Ferguson EL (2008) An interactive 24-hour recall for assessing the adequacy of iron and zinc intakes in developing countries. *HarvestPlus Technical Monograph 8*. Washington, DC and Cali: International Center for Tropical Agriculture (CIAT).
31. WHO FAO. Edited by Allen L, de Benoist B, Dary O & Hurrell R (2006) *Guidelines on food fortification with micronutrients*.
32. Institute of Medicine (2006) *Dietary Reference Intakes: The Essential Guide to Nutrient Requirements*. Washington, DC: The National Academies Press. <https://doi.org/10.17226/11537>. (accessed July 2024).
33. FAO/WHO (Food and Agriculture Organization/World Health Organization) (2002) *Human vitamin and mineral requirements*. Food and Nutrition Division, Food and Agriculture Organization, Rome.
34. Steyn NP, Nel J, Labadarios D *et al.* (2014) Which dietary diversity indicator is best to assess micronutrient adequacy in children 1 to 9 y? *Nutrition* **30**, 55–60.
35. Torrico JC (2021) Dietary diversity score as an indicator of micronutrient intake in Filipino children and adolescents. *Asia Pacific Journal of Clinical Nutrition*. **30**(4), 696-703.
36. Monge-Rojas R, Vargas-Quesada R, Marrón-Ponce JA *et al*. (2024) Exploring Differences in Dietary Diversity and Micronutrient Adequacy between Costa Rican and Mexican Adolescents. *Children.* **11**, 64. https://doi.org/10.3390/children11010064.
